# Prevention of age‐related neuromuscular junction degeneration in sarcopenia by low‐magnitude high‐frequency vibration

**DOI:** 10.1111/acel.14156

**Published:** 2024-03-27

**Authors:** Zhengyuan Bao, Can Cui, Chaoran Liu, Yufeng Long, Ronald Man Yeung Wong, Senlin Chai, Ling Qin, Clinton Rubin, Benjamin Hon Kei Yip, Zhihong Xu, Qing Jiang, Simon Kwoon‐Ho Chow, Wing‐Hoi Cheung

**Affiliations:** ^1^ Musculoskleletal Research Laboratory, Department of Orthopaedics and Traumatology, Prince of Wales Hospital The Chinese University of Hong Kong Hong Kong SAR China; ^2^ Division of Sports Medicine and Adult Reconstructive Surgery, Department of Orthopedic Surgery, Nanjing Drum Tower Hospital, Affiliated Hospital of Medical School Nanjing University Nanjing Jiangsu China; ^3^ Department of Biomedical Engineering Stony Brook University Stony Brook New York USA; ^4^ School of Public Health and Primary Care, Faculty of Medicine The Chinese University of Hong Kong Hong Kong SAR China; ^5^ Department of Orthopaedic Surgery Stanford University Stanford California USA; ^6^ Li Ka Shing Institute of Health Sciences The Chinese University of Hong Kong Hong Kong SAR China

**Keywords:** Dok7, ERK1/2, neuromuscular junction, sarcopenia, vibration

## Abstract

Neuromuscular junction (NMJ) degeneration is one of pathological factors of sarcopenia. Low‐magnitude high‐frequency vibration (LMHFV) was reported effective in alleviating the sarcopenia progress. However, no previous study has investigated treatment effects of LMHFV targeting NMJ degeneration in sarcopenia. We first compared morphological differences of NMJ between sarcopenic and non‐sarcopenic subjects, as well as young and old C57BL/6 mice. We then systematically characterized the age‐related degeneration of NMJ in SAMP8 against its control strain, SAMR1 mice, from 3 to 12 months old. We also investigated effects of LMHFV in SAMP8 on the maintenance of NMJ during the onset of sarcopenia with respect to the Agrin‐LRP4‐MuSK‐Dok7 pathway and investigated the mechanism related to ERK1/2 signaling. We observed sarcopenic/old NMJ presented increased acetylcholine receptors (AChRs) cluster fragmentation and discontinuity than non‐sarcopenic/young NMJ. In SAMP8, NMJ degeneration (morphologically at 6 months and functionally at 8 months) was observed associated with the sarcopenia onset (10 months). SAMR1 showed improved NMJ morphology and function compared with SAMP8 at 10 months. Skeletal muscle performance was improved at Month 4 post‐LMHFV treatment. Vibration group presented improved NMJ function at Months 2 and 6 posttreatment, accompanied with alleviated morphological degeneration at Month 4 posttreatment. LMHFV increased Dok7 expression at Month 4 posttreatment. In vitro, LMHFV could promote AChRs clustering in myotubes by increasing Dok7 expression through suppressing ERK1/2 phosphorylation. In conclusion, NMJ degeneration was observed associated with the sarcopenia onset in SAMP8. LMHFV may attenuate NMJ degeneration and sarcopenia progression by increasing Dok7 expression through suppressing ERK1/2 phosphorylation.

## Graphical abstract

In the sarcopenic SAMP8 model, NMJ degeneration preceded the onset of sarcopenia. LMHFV attenuated NMJ degeneration and sarcopenia progression by increasing Dok7 expression through suppressing ERK1/2 phosphorylation in skeletal muscle.

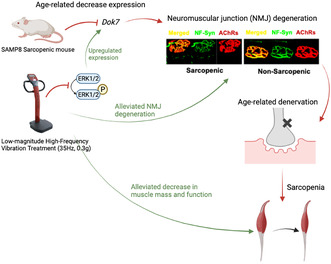

**Abbreviations**
AChE: acetylcholinesterase
AChRs: acetylcholine receptors
ASM: appendicular skeletal muscle mass
ASMI: appendicular skeletal muscle mass index
CDC: Centers for Disease Control and Prevention
Dok7: docking protein‐7
DXA: dual energy X‐ray absorptiometry
ERK: extracellular signal‐regulated kinase
LMHFV: low‐magnitude high‐frequency vibration
LRP4: low‐density lipoprotein receptor‐related protein 4
MAPKs: mitogen‐activated protein kinases
MuSK: muscle‐specific kinase
NMJ: neuromuscular junction
Rapsyn: receptor‐associated protein of the synapse
SAM: senescence‐accelerated mouse
SAMP: senescence‐prone inbred strains
SAMR: senescence‐resistant inbred strains

## 1 INTRODUCTION

Sarcopenia is a geriatric syndrome characterized by progressive decrease of muscle mass and strength (Cruz‐Jentoft et al., 2010). The etiology of sarcopenia is multifactorial with the degeneration of neuromuscular junction (NMJ) being one of the major causes (Rolland et al., 2008). Aging‐related changes in NMJ have been well reported to show morphological changes and functional adaptations (Deschenes et al., 2010). The maintenance of endplate morphology during aging is affected by acetylcholine receptors (AChRs) clustering at the postsynaptic cleft on the muscle side initiated by the large proteoglycan, Agrin, released at the presynaptic nerve endings (Bao et al., 2020). Across the muscle side, Agrin binds to the low‐density lipoprotein receptor‐related protein 4 (LRP4) to trigger the auto‐phosphorylation of the muscle‐specific kinase (MuSK) along with the substrate of docking protein‐7 (Dok7) that eventually leads to the clustering of AChRs through the receptor‐associated protein of the synapse (Rapsyn) (Burden et al., 2018).

Low‐magnitude high‐frequency vibration treatment (LMHFV, 35 Hz, 0.3 g) was found to be clinically effective in reducing fall incidences and enhancing muscle performance in community dwelling older people (Leung et al., 2014). A meta‐analysis of six studies revealed that vibration therapy was effective in improving muscle strength and physical performance in older adults with sarcopenia (Wu et al., 2020). This modality was also effective in maintaining skeletal muscle function in sarcopenic animal model with evidence to target other pathological factors like intramuscular fat infiltration (Wang et al., 2020). LMHFV has also been recently recommended by Centers for Disease Control and Prevention (CDC) to be an effective fall prevention intervention for elderly and published in the 4th Edition of Compendium (Burns et al., 2023). However, there is no previous study investigating the treatment effects of LMHFV specifically targeting denervation in sarcopenia.

The senescence‐accelerated mouse (SAM) was developed through selective inbreeding of the AKR/J strain based on a graded score for senescence, life span, and pathologic phenotype (Takeda et al., 1997). SAM strains contain senescence‐prone inbred strains (SAMP) and senescence‐resistant inbred strains (SAMR). SAMP strains are characterized with accelerated senescence and aging‐associated pathologies, while SAMR strains show a normal aging process. SAMP8 mouse is commonly used as an age‐associated sarcopenia model. Our previous study reported that the peak of muscle mass and strength appeared at 8 months old, followed by a significant decline at 10 months old in SAMP8 mice (Guo et al., 2015).

This study was conducted to characterize NMJ changes in sarcopenic patients, the age‐related degeneration of NMJ in C57BL/6 mice and SAMP8 mice against its control strain, SAMR1 mice (Takeda et al., 1997; Wang et al., 2023). Then we investigated the treatment effects of LMHFV therapy on the maintenance of the NMJ during the onset of sarcopenia in SAMP8 with respect to the Agrin‐LRP4‐MuSK‐Dok7‐Rapsyn pathway and extracellular signal‐regulated kinase (ERK)1/2 signaling.

## 2 RESULTS

### 2.1 Deterioration of NMJ morphology in sarcopenic versus non‐sarcopenic human subjects and young versus old C57BL/6 mice

**FIGURE 1:**
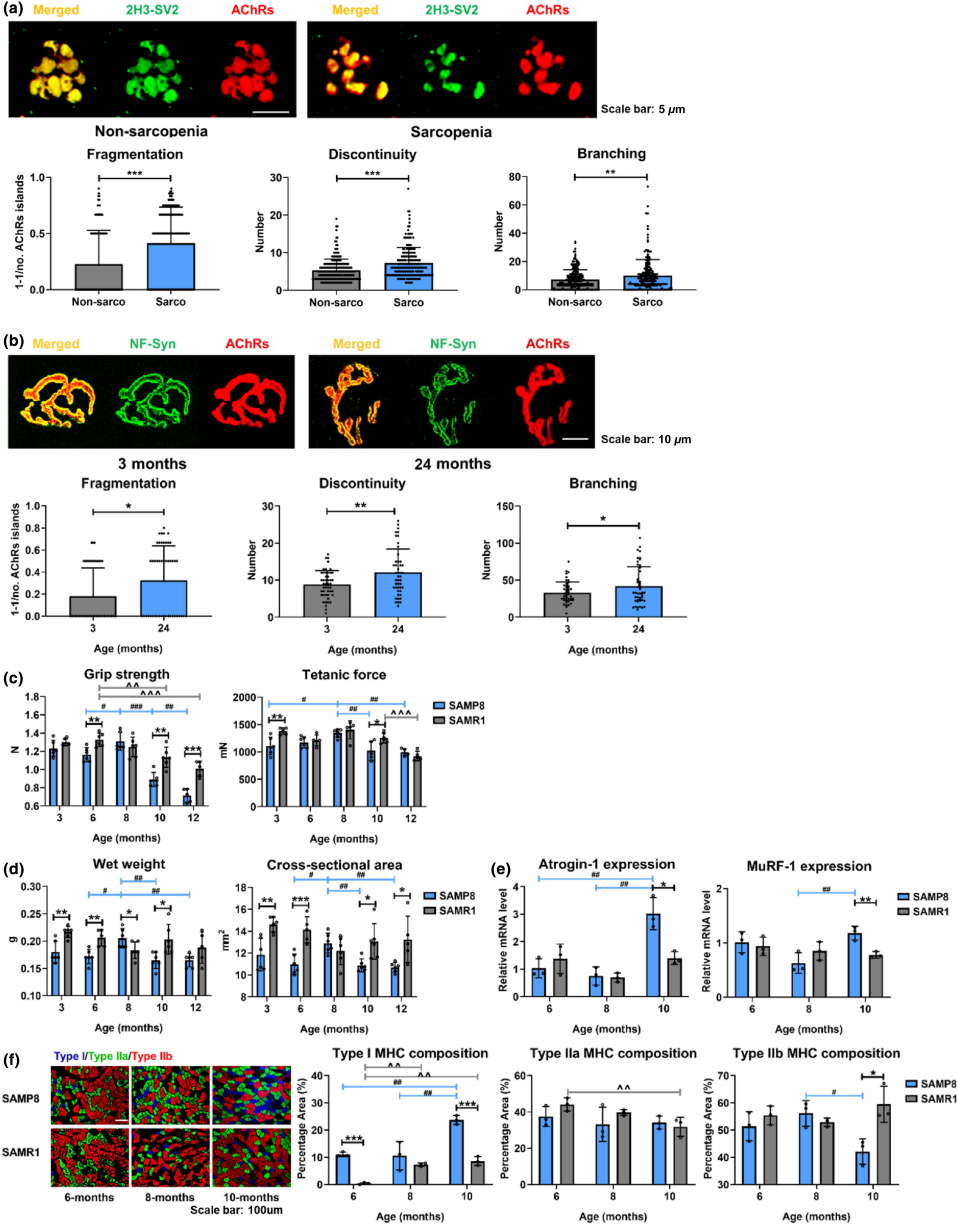
Morphological characteristics of NMJ in sarcopenic subjects and skeletal muscle degeneration in terms of muscle function, mass, and gene expression in sarcopenic mice. (a) Fragmentation (*F* for variance = 0.389, *p* = 0.533), discontinuity (*F* for variance = 16.357, *p* < 0.05), and branching (*F* for variance = 13.488, *p* < 0.05) assessment from NMJ staining images of vastus lateralis muscle in sarcopenic (*n* = 5, 200 NMJs, minimum 35 NMJs in each patient) and non‐sarcopenic patients (*n* = 4, 230 NMJs, minimum 43 NMJs in each patient). (b) Fragmentation (*F* for variance = 10.446, *p* < 0.05), discontinuity (*F* for variance = 10.66, *p* < 0.05), and branching (*F* for variance = 16.566, *p* < 0.05) assessment from NMJ staining images of extensor digitorum longus muscle in Month 3 (*n* = 3, 47 NMJs, minimum 12 NMJs in each mouse) and 24 C57BL/6 mice (*n* = 3, 46 NMJs, minimum 10 NMJs in each mouse). (c) Grip strength (*n* = 6) and ex vivo triceps surae muscle tetanic force (*n* = 5–6) of SAMP8 and SAMR1 at 3, 6, 8, and 10 months old. (d) Wet weight and cross‐sectional area (*n* = 5–6) of triceps surae muscle of SAMP8 and SAMR1 at 3, 6, 8, and 10 months old. (e) Atrogin‐1 and MuRF‐1 mRNA expressions in extensor digitorum longus muscle of SAMP8 and SAMR1 at 6, 8, and 10 months (*n* = 3). (f) Muscle fiber MHC composition of gastrocnemius muscle in SAMP8 and SAMR1 at 6, 8, and 10 months (*n* = 3). (2H3, neurofilament antibody; SV2, synaptic vesicle glycoprotein 2A antibody; NF, neurofilament; Syn, synaptophysin). Error bars represent the SD of the mean. Statistical significance was determined using Student's *t*‐test (a, b) and Bonferroni or Tamhane's T2 post hoc test following one‐way ANOVA accompanied with Student's *t*‐test (c–f). *p < 0.05, **p < 0.01, ***p < 0.001 for Student's *t*‐test. ^#^ *p* < 0.05, ^##^ *p* < 0.01, ^###^ *p* < 0.001 for one‐way ANOVA of SAMP8 mice. ^^^^ *p* < 0.01, ^^^^^ *p* < 0.001 for one‐way ANOVA of SAMR1 mice.

Sarcopenic patients (4 female and 1 male, 81 ± 8 years) presented reduced grip strength and ASMI (14.40 ± 3.21 kg vs. 22.50 ± 4.20 kg, *p* < 0.05 and 4.39 ± 0.8 kg/m^2^ vs. 5.97 ± 1.07 kg/m^2^, respectively) than non‐sarcopenic patients (4 female, 74 ± 8 years) (Table S1). Sarcopenic NMJ presented increased AChRs cluster fragmentation (0.41 ± 0.32 vs. 0.23 ± 0.30, *p* < 0.001), discontinuity (7.24 ± 4.15 vs. 5.30 ± 2.97, *p* < 0.001), and branching number (10.05 ± 11.30 vs. 7.33 ± 6.84, *p* < 0.01) than non‐sarcopenic NMJ (Figure 1a).

C57BL/6 mice aged 24 months old showed increased AChRs cluster fragmentation (0.33 ± 0.31 vs. 0.18 ± 0.26, *p* < 0.05), discontinuity (12.13 ± 6.32 vs. 8.87 ± 3.75, *p* < 0.01), and branching number (42.09 ± 25.87 vs. 33.15 ± 14.31, *p* < 0.05) compared with 3 months (Figure 1b). Taken together, development of sarcopenia showed a deterioration in NMJ morphology in humans and C57BL/6 mice that may require further investigations in terms of morphology and function.

### 2.2 SAMP8 was validated as sarcopenic animal model with SAMR1 as the age‐matched control

In order to investigate NMJ degeneration in relation to the onset of sarcopenia, a well‐accepted sarcopenic animal model, SAMP8 against its control strain, SAMR1 without sarcopenia phenotype were used in this study (Wang et al., 2023). In SAMP8, grip strength peaked at 8 months and decreased significantly by 32.00% at 10 months (*p* < 0.001). In SAMR1, grip strength also showed significant 14.54% decrease at 10 months compared with 6 months (*p* < 0.01), but SAMR1 significantly presented 27.35% and 41.17% higher grip strength than SAMP8 at 10 months and 12 months (*p* < 0.01 and <0.001, respectively) (Figure 1c). Tetanic force of SAMP8 also peaked at 8 months followed by a significant decrease at 10 months (*p* < 0.01). At 10 months, SAMR1 mice presented 22.94% higher tetanic force than SAMP8 (*p* < 0.05) (Figure 1c). Muscle mass increased from 6 to 8 months but dropped significantly by 19.51% of wet weight and 15.63% of cross‐sectional area (CSA) at 10 months in SAMP8 (wet weight: *p* < 0.05 and <0.01, respectively; CSA: *p* < 0.05 and <0.01, respectively). At 10 months, SAMR1 presented larger wet weight and CSA than SAMP8 (*p* < 0.05 for both). At 12 months, SAMR1 presented larger CSA than SAMP8 (*p* < 0.05) (Figure 1d). Furthermore, mRNA levels of muscle atrophy markers, Atrogin‐1 and MuRF‐1, were significantly increased from 8 to 10 months in SAMP8 (*p* < 0.01 for both). And at 10 months, SAMR1 presented significantly lower mRNA expressions of Atrogin‐1 and MuRF‐1 than SAMP8 (*p* < 0.05 and <0.01, respectively) (Figure 1e). SAMP8 presented a significant 124.09% higher expression of Type I and 25.01% lower expression of Type IIb fibers from 8 to 10 months (*p* < 0.01 and <0.05, respectively). SAMR1 was also shown to exhibit a significantly 63.69% lower proportion of Type I but 41.23% higher proportion of Type IIb fibers than SAMP8 at 10 months (*p* < 0.001 and <0.05, respectively) (Figure 1f). Taken together, sarcopenic phenotype of SAMP8 was confirmed at 10 months of age against its SAMR1 control strain.

### 2.3 Morphological and functional degeneration of NMJ was associated with the onset of sarcopenia in sarcopenic mice

**FIGURE 2:**
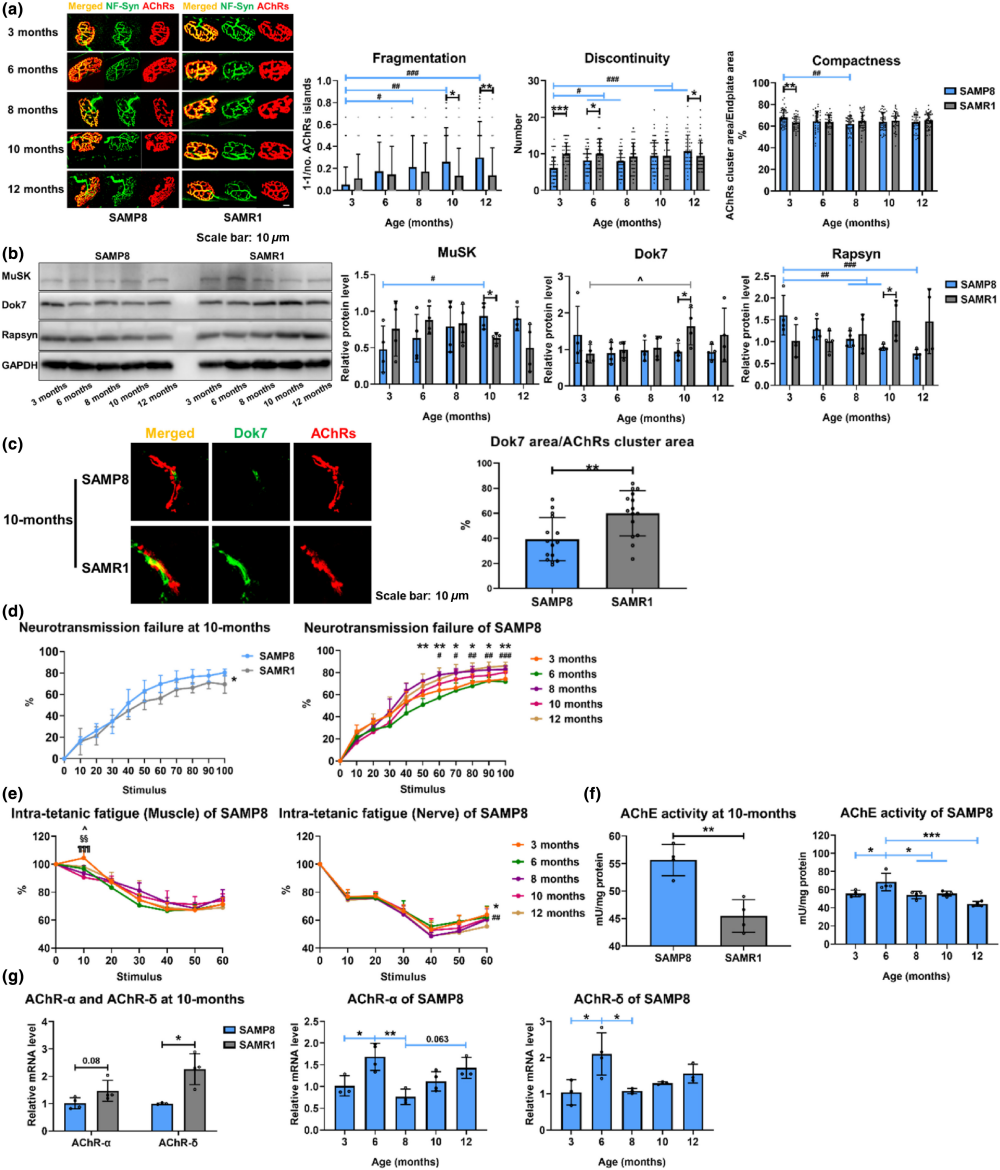
Morphological and functional degeneration of NMJ in sarcopenic mice. (a) Fragmentation, discontinuity, and compactness assessment from NMJ staining images of extensor digitorum longus muscle in SAMP8 and SAMR1 at 3, 6, 8, 10, and 12 months old (*n* = 41–51 NMJs in 5–6 mice, minimum 7 NMJs in each mouse). (b) Western blots results of MuSK, Dok7, Rapsyn, and GAPDH in SAMP8 and SAMR1 tibialis anterior muscle at 3, 6, 8, 10, and 12 months old (*n* = 4). (c) Assessment of compactness between Dok7 staining area and AChRs cluster area in SAMP8 and SAMR1 gastrocnemius muscle at 10 months old (*n* = 15 clusters in three mice). (d) Neurotransmission failure of triceps surae‐sciatic nerve in SAMP8 and SAMR1 at 10 months old and SAMP8 at 3, 6, 8, 10, and 12 months old (*n* = 5–6). (e) Intra‐tetanic fatigue of triceps surae‐sciatic nerve in SAMP8 at 3, 6, 8, 10, and 12 months old (*n* = 5–6). (f) AChE activity of tibialis anterior muscle in SAMP8 and SAMR1 at 10 months and SAMP8 at 3, 6, 8, 10, and 12 months (*n* = 4). (g) mRNA expressions of AChR‐α and ‐δ subunit in SAMP8 and SAMR1 extensor digitorum longus muscle at 10 months and SAMP8 at 3, 6, 8, 10, and 12 months (*n* = 3–4). (NMJ: neuromuscular junction; AChRs: acetylcholine receptors; AChE: acetylcholinesterase). Error bars represent the SD of the mean. Statistical significance was determined using Bonferroni, LSD or Tamhane's T2 post hoc test following one‐way ANOVA accompanied with Student's *t*‐test (a, b), Student's *t*‐test (c, f *left* and g *left*), Bonferroni post hoc test following two‐way repeated measures ANOVA (d, e), Bonferroni post hoc test following one‐way ANOVA (f *right* and g *right two*). *p < 0.05, **p < 0.01, ***p < 0.001 for Student's *t*‐test (a–c, f *left* and g *left*), two‐way repeated measures ANOVA (d *left* for SAMP8 vs. SAMR1, d *right* and e *right* for 6 vs. 8 months old), or one‐way ANOVA (f *right* and g *right two*). ^#^ *p* < 0.05, ^##^ *p* < 0.01, ^###^ *p* < 0.001 for one‐way ANOVA of SAMP8 mice (a, b), two‐way repeated measures ANOVA (d *right* and e *right* for 6 vs. 12 months old). ^^^ *p* < 0.05 for one‐way ANOVA of SAMR1 mice (b), two‐way repeated measures ANOVA (e *left* for 3 vs. 6 months old). ^§§^ *p* < 0.01 for two‐way repeated measures ANOVA (e *left* for 3 vs. 8 months old). ^¶¶¶^ *p* < 0.001 for two‐way repeated measures ANOVA (e *left* for 3 vs. 10 months old).

To investigate if NMJ degeneration was associated with the onset of sarcopenia, NMJ morphology was further investigated. SAMP8 presented significantly increased AChRs cluster fragmentation and discontinuity from 3 to 12 months (*p* < 0.001 for both), while SAMR1 presented no significant differences with increasing age. At 10 and 12 months, SAMR1 showed significantly reduced cluster fragments (*p* < 0.05 and <0.01, respectively) and discontinuity (*p* < 0.05 at 12 months) compared with SAMP8 (Figure 2a). Compactness between AChRs cluster and endplate area was significantly reduced from 3 to 8 months in SAMP8 (*p* < 0.01) (Figure 2a), indicating a process of AChRs cluster dispersion in sarcopenia.

SAMP8 presented reduced Rapsyn protein expressions with aging (Figure 2b). The gradually increased MuSK could be taken as a compensatory mechanism for reduced Rapsyn (Figure 2b). In SAMR1, MuSK protein was expressed significantly lower than in SAMP8 at 10 months (*p* < 0.05) (Figure 2b). Both Dok7 and Rapsyn proteins increased with aging in SAMR1 and were significantly overexpressed versus SAMP8 at 10 months (*p* < 0.05 for both) (Figure 2b). Furthermore, the compactness between Dok7 staining area and AChRs cluster was larger in SAMR1 than in SAMP8 at 10 months (*p* < 0.01) (Figure 2c). Taken together, morphological degeneration of NMJ was found to happen around 6 months old in SAMP8, preceding the occurrence of sarcopenia at 10 months. Besides, Dok7 was found to exhibit a difference between sarcopenic SAMP8 and non‐sarcopenic SAMR1.

We further investigated the NMJ functional changes during the onset of sarcopenia. At 10 months, SAMP8 presented significantly increased neurotransmission failure (NF) than SAMR1 (*p* < 0.05) (Figure 2d). In SAMP8 with increasing age, NF was significantly increased at 8 months compared with 6 months (*p* < 0.05 at 70th, 80th, and 90th stimulus and *p* < 0.01 at 50th, 60th, and 100th stimulus), yet no significant difference was found between 8 and 12 months (Figure 2d), indicating a remarkable deterioration of neurotransmission at 8 months in SAMP8. Intra‐tetanic fatigue (IF) was another parameter assessing NMJ function and given by the percentage force at the end of every 10 stimuli compared to the maximum force generated during that same pulse train. SAMP8 at 8 months presented no significant difference of IF after direct muscle stimulations compared with 6 months, but after nerve stimulations, IF at 8 months significantly decreased compared with 6 months (*p* < 0.05) (Figure 2e). Furthermore, from 8 to 12 months old, no significant differences were observed in IF of both muscle and nerve stimulations in SAMP8 (Figure 2e). These results suggested significant NMJ fatigue at 8 months in SAMP8.

To further investigate the mechanisms of NMJ functional degeneration in SAMP8, AChE activity was measured. At 10 months, SAMR1 showed significantly reduced AChE activity than SAMP8 (*p* < 0.01). AChE activity significantly increased from 3 to 6 months followed by a significant reduction at 8 months compared with 6 months (*p* < 0.05 for both) (Figure 2f).

AChRs are formed by five subunits in which α and δ subunits constitute the binding site with acetylcholine (ACh) (Green & Wanamaker, 1998). At 10 months, SAMR1 showed increased mRNA expression of α and δ subunits than SAMP8 (*p* = 0.08 and <0.05, respectively). mRNA expressions of both α and δ subunits in SAMP8 were significantly increased from 3 to 6 months, followed by a reduction at 8 months compared with 6 months (α subunit: *p* < 0.05 and <0.01, respectively; δ subunit: *p* < 0.05 for both) (Figure 2g). Therefore, functional degeneration of NMJ was accompanied with a significant drop of AChRs structural components and AChE activities in sarcopenic SAMP8 at 8 months. This degeneration was associated with the onset of sarcopenia at 10 months.

### 2.4 LMHFV attenuated NMJ degeneration in sarcopenic mice

**FIGURE 3:**
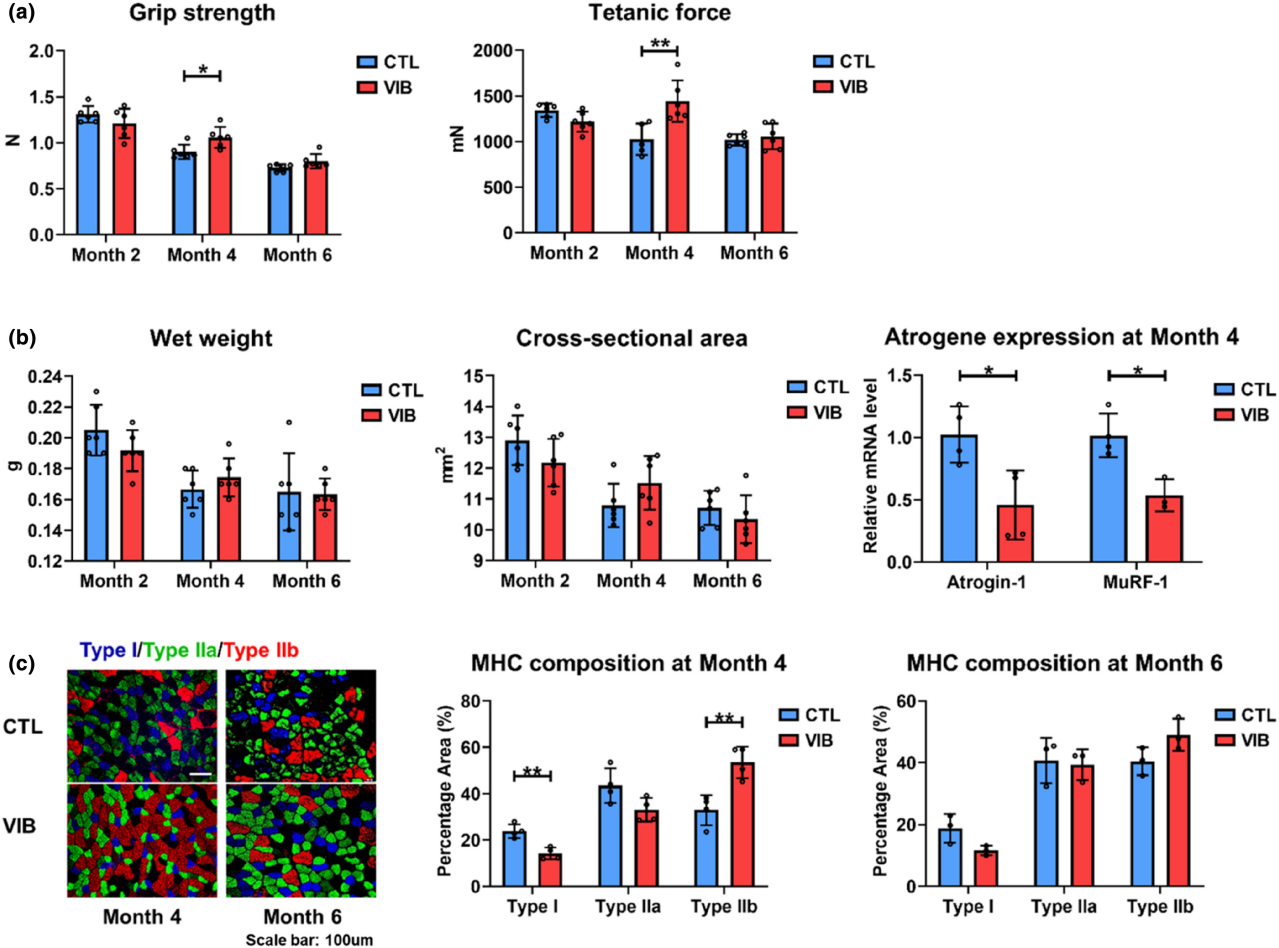
Skeletal muscle degeneration was suppressed by vibration treatment in SAMP8 mice. (a) Grip strength (*n* = 6) and ex vivo triceps surae muscle tetanic force (*n* = 5–6) of SAMP8 at Months 2, 4, and 6 posttreatment (8, 10, and 12 months old). (b) Wet weight and cross‐sectional area (*n* = 6) of triceps surae muscle at Months 2, 4, and 6 posttreatment in SAMP8 (8, 10, and 12 months old). Atrogin‐1 and MuRF‐1 mRNA levels of extensor digitorum longus muscle at Month 4 posttreatment in SAMP8 (10 months old, *n* = 3–4). (c) Muscle fiber MHC composition of gastrocnemius muscle in SAMP8 at Months 4 and 6 posttreatment (10 and 12 months old, *n* = 3–4). (CTL, control group; g, grams; MHC, myosin heavy chain; mN, milliNewtons; N, Newtons; VIB, vibration group). Error bars represent the SD of the mean. Statistical significance was determined using Student's *t*‐test (a–c). *p < 0.05, **p < 0.01.

As a well‐investigated biophysical intervention, LMHFV was provided as a treatment to investigate its effects on sarcopenia. At Month 4 posttreatment, both grip strength and triceps surae muscle tetanic force were elevated in VIB group compared with CTL group (*p* < 0.05 and <0.01, respectively). At Month 6 posttreatment, LMHFV showed no more significant effects on both grip strength and triceps surae muscle tetanic force (Figure 3a). Although LMHFV presented no significant effects on muscle mass, VIB group showed significantly reduced mRNA expressions of Atrogin‐1 and MuRF‐1 at Month 4 posttreatment (*p* < 0.05 for both) (Figure 3b). Furthermore, LMHFV could significantly reduce Type I fiber distribution by 40.13% but increase Type IIb fiber composition by 62.71% at Month 4 posttreatment (*p* < 0.01 for both). But at Month 6 posttreatment, LMHFV showed no significant effects on MHC composition (Figure 3c). Taken together, LMHFV could significantly improve muscle quality in sarcopenic SAMP8, yet it had no substantial impact on muscle mass, which may be attributable to changes in MHC fiber composition.

**FIGURE 4:**
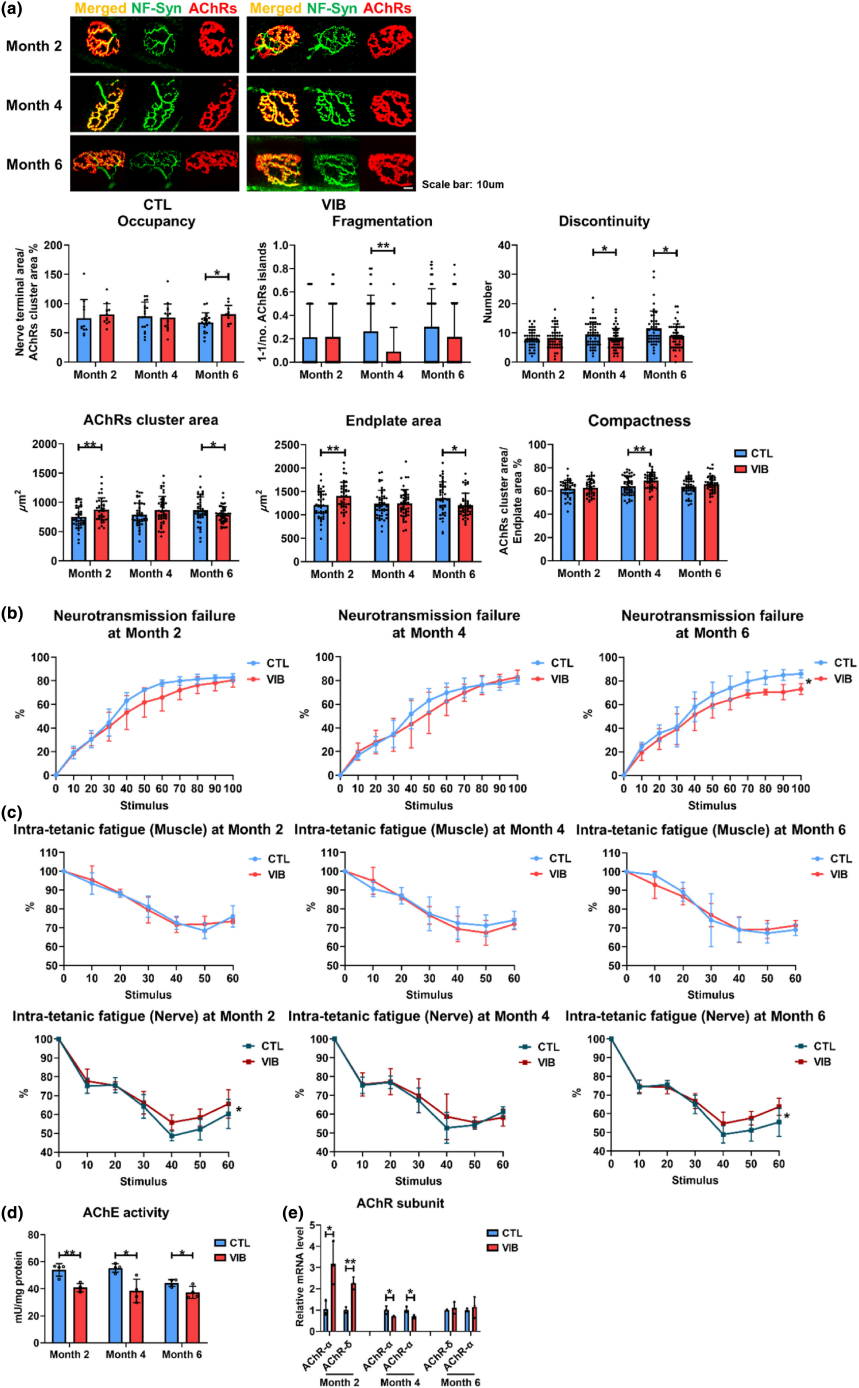
Vibration treatment alleviated NMJ morphological and functional deterioration in SAMP8 mice. (a) Occupancy, fragmentation, discontinuity, AChRs cluster area, endplate area, and compactness assessment from NMJ staining images of extensor digitorum longus muscle in SAMP8 at Months 2, 4, and 6 posttreatment (8, 10, and 12 months old, *n* = 11–48 NMJs in 5–6 mice, for occupancy, minimum 2 NMJs in each mouse, for fragmentation, discontinuity, AChRs cluster area, endplate area, and compactness, minimum 7 NMJs in each mouse). (b) Neurotransmission failure of triceps surae‐sciatic nerve in SAMP8 at Months 2, 4, and 6 posttreatment (8, 10, and 12 months old, *n* = 5–6). (c) Intra‐tetanic fatigue of triceps surae‐sciatic nerve in SAMP8 at Months 2, 4, and 6 posttreatment (8, 10, and 12 months old, *n* = 5–6). (d) AChE activity of tibialis anterior muscle in SAMP8 at Month 2, 4, and 6 posttreatment (8, 10, and 12 months old, *n* = 4). (e) AChR‐α and δ subunit mRNA expressions of extensor digitorum longus muscle in SAMP8 at Months 2, 4, and 6 posttreatment (8, 10, and 12 months old, *n* = 3). (AChE, acetylcholinesterase; AChRs, acetylcholine receptors; CTL, control group; NMJ, neuromuscular junction; VIB, vibration group). Error bars represent the SD of the mean. Statistical significance was determined using Student's *t*‐test (a, d, e), Bonferroni post hoc test following two‐way repeated measures ANOVA (b, c). *p < 0.05, **p < 0.01.

To further investigate the treatment effect of LMHFV on the preservation of NMJ, LMHFV was given to SAMP8 at 6 months of age (the onset of NMJ mophological degeneration at 6 months). At Month 4 posttreatment, cluster fragmentation and discontinuity was significantly alleviated (*p* < 0.01 and <0.05, respectively) (Figure 4a). Although LMHFV presented no significant effects on AChRs cluster area and endplate area at Month 4 posttreatment, the compactness was significantly increased (*p* < 0.01) (Figure 4a), indicating alleviated AChRs dispersion. At Month 6 posttreatment, AChRs cluster innervation and discontinuity was significantly improved (*p* < 0.05 for both) (Figure 4a). Taken together, morphological degeneration of NMJ that was mainly alleviated at Months 4 and 6 posttreatment.

We also explored the effects of LMHFV on NMJ function. VIB group presented significantly reduced NF at Month 6 posttreatment (*p* < 0.05), yet no difference at Months 2 and 4 posttreatment (Figure 4b). The assessment of NMJ fatigue revealed that LMHFV presented no significant effects on the IF after direct muscle stimulations, but at Months 2 and 6 posttreatment, IF was significantly improved after nerve stimulations (*p* < 0.05 for both) (Figure 4c), indicating LMHFV presented no significant effects on muscle fatigue but could significantly attenuate NMJ fatigue.

To further study the mechanisms of improved NMJ function, it was found that LMHFV could significantly reduce AChE activity at Months 2, 4, and 6 posttreatment (*p* < 0.01, <0.05, and <0.05, respectively) (Figure 4d). This could be explained by that the neuromuscular system would be recruited to generate tetanic and isometric contraction force during LMHFV treatment, while reduced AChE activity could ensure sufficient binding between ACh and AChRs. Furthermore, α and δ subunit expressions were significantly increased at Month 2 posttreatment (*p* < 0.05 and <0.01, respectively) (Figure 4e), altogether contributing to the binding between ACh and AChRs, thus resulting in improved NMJ function at Month 2 posttreatment. All these results indicated that LMHFV could alleviate NMJ functional degeneration in a short term by reducing AChE activity and increasing α and δ subunit expression. And the alleviated NMJ morphological degeneration and reduced AChE activity could be the reason that led to improved NMJ function at Month 6 posttreatment in SAMP8 mice.

### 2.5 LMHFV promoted AChRs clustering and attenuated muscle atrophy by increasing Dok7

**FIGURE 5:**
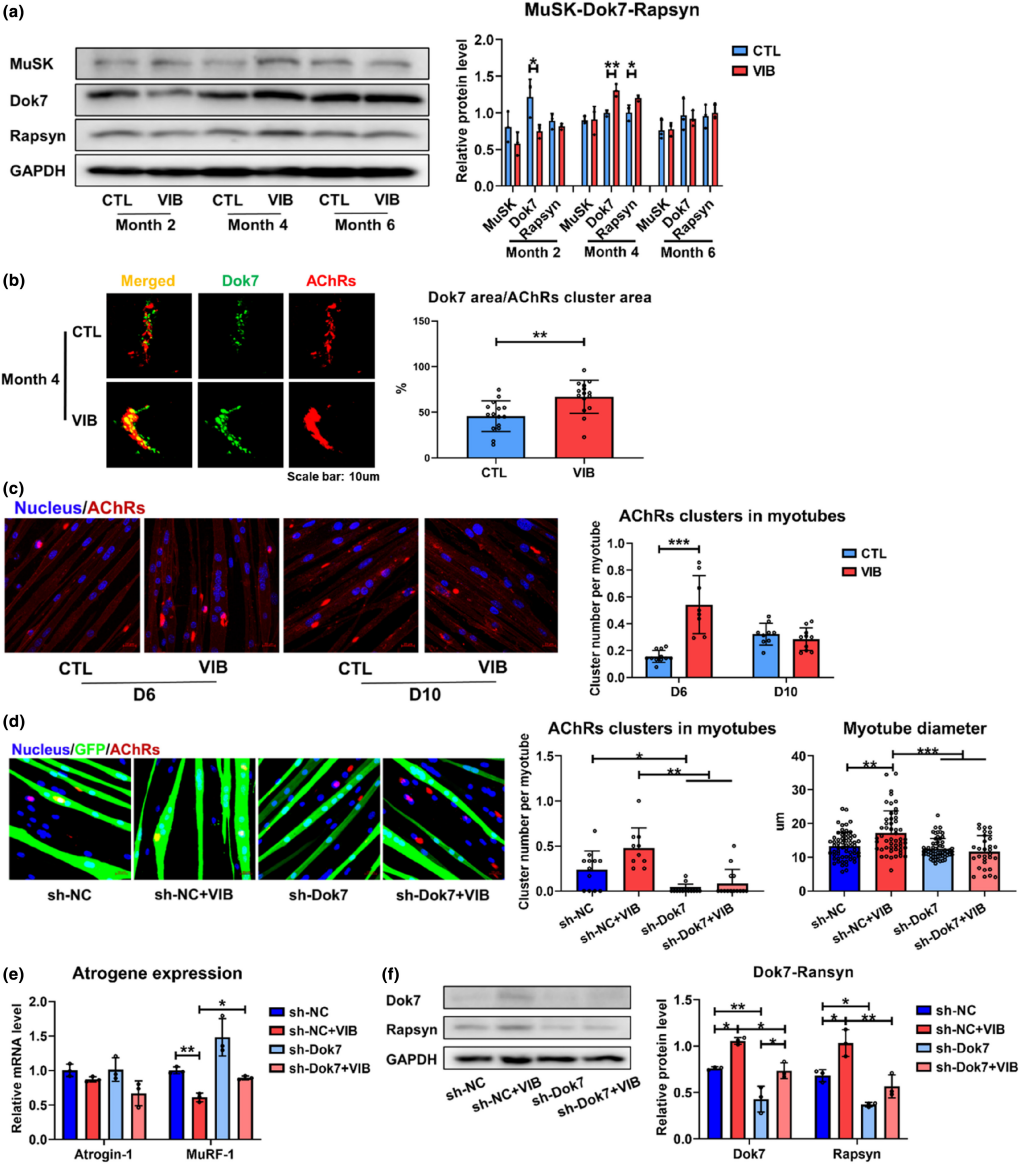
LMHFV enhanced AChRs clustering by increasing Dok7 expression. (a) Western blot results of MuSK, Dok7, Rapsyn, and GAPDH of tibialis anterior muscle in SAMP8 at Months 2, 4, and 6 posttreatment (8, 10, and 12 months old, *n* = 3). (b) Assessment of compactness between Dok7 staining area and AChRs cluster area of gastrocnemius muscle at Month 4 posttreatment (10 months old, *n* = 15 clusters in three mice). (c) AChRs cluster (>10 μm^2^) formation at D6 (LMHFV was applied from D1 to D6) and D10 (LMHFV was applied from D5 to D10) of myoblast differentiation (*n* = 8–11 fields). (d) AChRs cluster (>10 μm^2^) formation (*n* = 10–14 fields, f for variance = 4.418, *p* < 0.05) and myotube diameter (*n* = 30–61 myotubes, f for variance = 10.097, *p* < 0.05) at D6 of myoblast differentiation in sh‐NC, sh‐NC + VIB, sh‐Dok7, and sh‐Dok7 + VIB group. (e) mRNA expressions of Atrogin‐1 and MuRF‐1 at D6 of myoblast differentiation in sh‐NC, sh‐NC + VIB, sh‐Dok7, and sh‐Dok7 + VIB group (*n* = 3). (f) Western blot results of Dok7, Rapsyn and GAPDH at D6 of myoblast differentiation in sh‐NC, sh‐NC + VIB, sh‐Dok7 and sh‐Dok7 + VIB group (*n* = 3). (AChRs, acetylcholine receptors; CTL, control group; LMHFV, low‐magnitude high‐frequency vibration; sh‐Dok7 + VIB, Dok7 knocking down + vibration group; sh‐Dok7, Dok7 knocking down group; sh‐NC + VIB, negative control + vibration group; sh‐NC, negative control group; VIB, vibration group). Error bars represent the SD of the mean. Statistical significance was determined using Student's *t*‐test (a–c), Bonferroni or Tamhane's T2 post hoc test following one‐way ANOVA (d–f). *p < 0.05, **p < 0.01, ***p < 0.001.

As LMHFV was observed to be effective in maintaining morphological integrity and thus the function of NMJ during the onset of sarcopenia, we further investigated the molecular mechanisms. As shown in Figure S5, mRNA expression of Dok7 was significantly increased at Month 4 posttreatment (*p* < 0.05). VIB group presented significantly increased Dok7 and Rapsyn protein expressions at Month 4 posttreatment (*p* < 0.01 and <0.05, respectively) (Figure 5a). Furthermore, VIB group showed larger compactness between Dok7 stained area and AChRs cluster area at Month 4 posttreatment (*p* < 0.01) (Figure 5b), indicating Dok7 may be the key factor for the effects of LMHFV on NMJ.

To validate the role of Dok7, primary myoblasts were isolated from SAMP8 mice. The time point of inducing differentiation was regarded as Day 0 (D0) and mature myotubes were observed to be formed at D5 (Figure S6A). LMHFV was then applied in two different schemes: D1 to D6 or D5 to D10 (Figure S6B). It was found that LMHFV could significantly increase the formation of large AChRs cluster (>10 μm^2^) when applied from D1 to D6 but presented no effects on cluster formation when applied from D5 to D10 (*p* < 0.001) (Figure 5c), indicating LMHFV functioned at the myoblast differential phase in mediating AChRs cluster formation. In later in vitro experiments, LMHFV was applied from D1 to D6 only. Dok7 was then knocked down by sh‐RNA transfection and primary myoblasts were then divided into four groups: negative control (sh‐NC), Dok7 knockdown (sh‐Dok7), sh‐NC + vibration group (sh‐NC + VIB), and sh‐Dok7 + vibration group (sh‐Dok7 + VIB). Compared with sh‐NC group, sh‐NC + VIB group presented larger myotube diameter (13.35 ± 4.08 μm vs. 17.26 ± 6.44 μm, *p* < 0.01). Knocking down Dok7 significantly suppressed AChRs cluster formation (0.23 ± 0.21 vs. 0.02 ± 0.05, *p* < 0.05), which could not be retarded by LMHFV (Figure 5d). LMHFV could also significantly decrease the mRNA expression of MuRF‐1 in vitro (*p* < 0.01) (Figure 5e). At the protein level, LMHFV significantly increased Dok7 and Rapsyn (*p* < 0.05 for both). Knocking down Dok7 significantly reduced Rapsyn level (*p* < 0.05) and LMHFV could not retard the reduction in myotubes with Dok7 knocked down. Dok7 protein expression in sh‐Dok7 + VIB group was significantly higher than that in sh‐Dok7 group, but still significantly lower than that in sh‐NC + VIB group (*p* < 0.05 for both), which may be attributed to transfection efficiency (Figure 5f). Taken together, LMHFV increased the expression of Dok7 at NMJ that contributed to the improvements in AChRs clustering and attenuation of muscle atrophy.

### 2.6 LMHFV increased Dok7 expression through suppressing ERK1/2 phosphorylation

**FIGURE 6:**
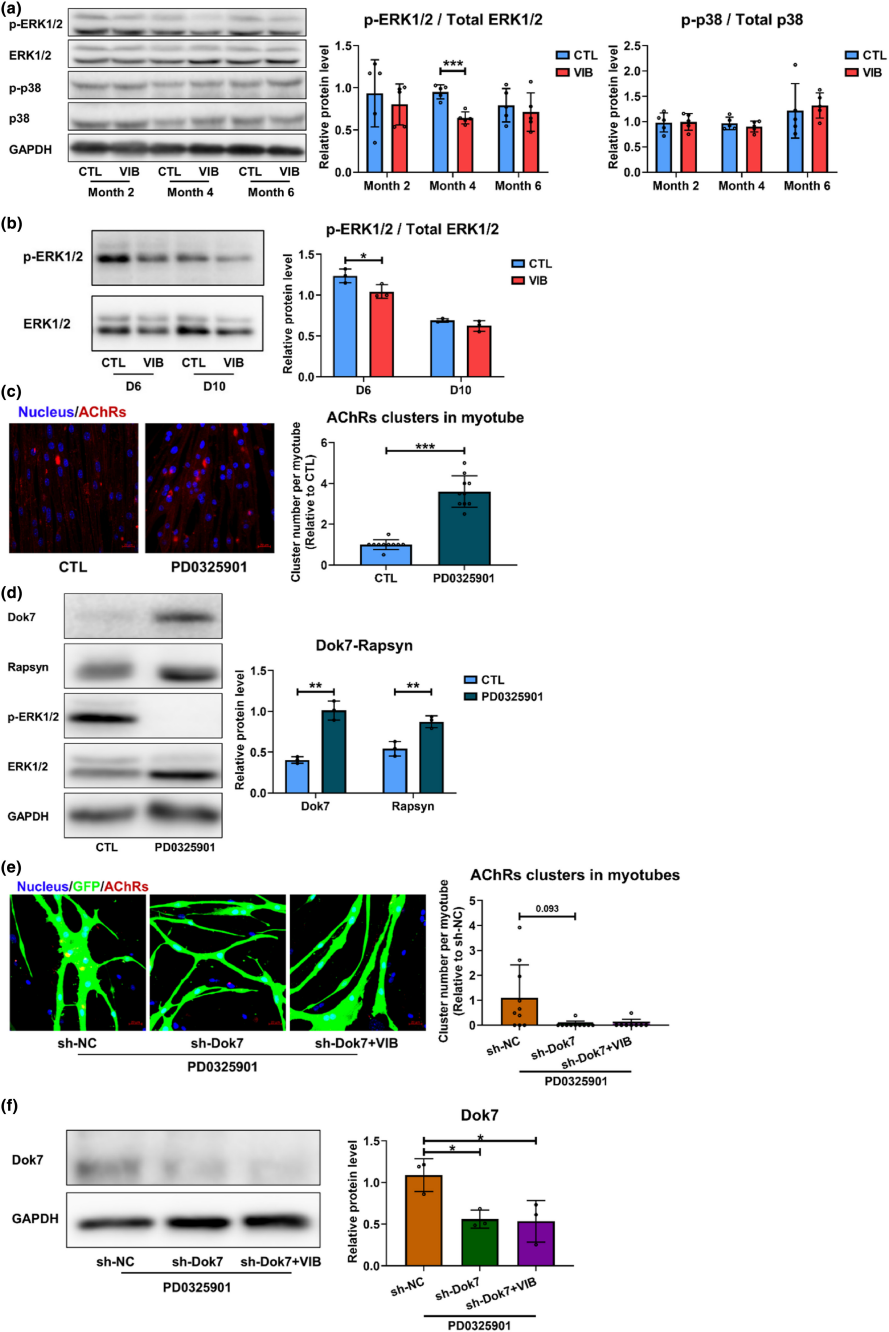
LMHFV increased expression of Dok7 by suppressing the activation of ERK1/2. (a) Western blot results of p‐ERK1/2, ERK1/2, p‐p38, p38, and GAPDH of tibialis anterior muscle in SAMP8 at month 2, 4 and 6 posttreatment (8, 10 and 12 months old, *n* = 5). (b) Western blot results of p‐ERK1/2, ERK1/2, and GAPDH at D6 (LMHFV was applied from D1 to D6) and D10 (LMHFV was applied from D5 to D10) of myoblast differentiation (*n* = 3). (c) AChRs cluster (>10 μm^2^) formation at D6 of myoblast differentiation when ERK1/2 phosphorylation was blocked (*n* = 10 fields). (d) Western blot results of Dok7, Rapsyn, p‐ERK, ERK and GAPDH at D6 of myoblast differentiation when ERK1/2 phosphorylation was blocked (*n* = 3). (e) AChRs cluster (>10 μm^2^) formation at D6 of myoblast differentiation in sh‐NC, sh‐Dok7 and sh‐Dok7 + VIB group when ERK1/2 phosphorylation was blocked (*n* = 8–10 fields, F for variance = 14.202, *p* < 0.001). (f) Western blot results of Dok7 and GAPDH at D6 of myoblast differentiation in sh‐NC, sh‐Dok7, and sh‐Dok7 + VIB group when ERK1/2 phosphorylation was blocked (*n* = 3). (CTL, control group; LMHFV, low‐magnitude high‐frequency vibration; sh‐Dok7 + VIB, Dok7 knocking down + vibration group; sh‐Dok7, Dok7 knocking down group; sh‐NC, negative control group; VIB, vibration group). Error bars represent the SD of the mean. Statistical significance was determined using Student's *t*‐test (a–d) or Bonferroni, Tamhane's T2 post hoc test following one‐way ANOVA (e, f). *p < 0.05, **p < 0.01, ***p < 0.001.

In skeletal muscles, mitogen‐activated protein kinases (MAPKs) family is composed of three distinct signaling modules: ERK1/2, p38, and JNKs (Zhao et al., 2015). MAPKs could transduce extracellular mechanical stress in an intensity‐dependent manner (Kramer & Goodyear, 2007). LMHFV significantly suppressed ERK1/2 phosphorylation at Month 4 posttreatment but presented no significant effects on p38 phosphorylation (*p* < 0.001) (Figure 6a). Besides, LMHFV significantly reduced ERK1/2 phosphorylation in sarcopenic myoblasts in vitro when applied from D1 to D6 of differentiation, yet no effects when applied from D5 to D10 (*p* < 0.05) (Figure 6b). These substantiated a previous study which reported that resistance exercise could reduce ERK1/2 phosphorylation in skeletal muscles of old men (Williamson et al., 2003). After inhibition of ERK1/2 phosphorylation in vitro, AChRs cluster formation was significantly increased (*p* < 0.001) (Figure 6c). As LMHFV could promote AChRs cluster formation when applied from D1 to D6 and ERK1/2 was reported to function in inhibiting myoblast differentiation (Knight & Kothary, 2011), our results suggested that LMHFV promoted myoblast differentiation by suppressing ERK1/2 phosphorylation.

To further validate the relationship between ERK1/2 and Dok7, it was found that inhibiting ERK1/2 phosphorylation significantly increased the protein expressions of Dok7 and Rapsyn in vitro (*p* < 0.01 for both) (Figure 6d), while knocking down Dok7 significantly suppressed ERK1/2 phosphorylation (*p* < 0.05) (Figure S8B). In myotubes with ERK1/2 phosphorylation inhibited, knocking down Dok7 still reduced AChRs cluster formation (*p* = 0.093) and significantly decreased Dok7 protein expression (*p* < 0.05), which could not be retarded by LMHFV (Figure 6e,f). Therefore, LMHFV increased Dok7 expression through suppressing ERK1/2 phosphorylation.

## 3 DISCUSSION

In this study, NMJ degeneration was found to be associated with the onset of sarcopenia in SAMP8, whereas SAMR1 presented better NMJ function and more intact NMJ morphology than sarcopenic SAMP8 at 10 months, indicating a possible causative relationship between NMJ degeneration and sarcopenia (Deschenes et al., 2010; Tintignac et al., 2015). During aging in SAMP8, no differences of NMJ function were observed between 3 and 6 and between 8 and 12 months old. However, SAMP8 presented impaired NMJ function from 6 to 8 months old, indicating NMJ functional degeneration in SAMP8 was abrupt and occurred rapidly (Li et al., 2011). NMJ function is related to neurotransmitter release, AChE activity and endplate excitability (Aldrich et al., 1986). From 6 to 8 months, the morphological degeneration of NMJ and decreased AChR‐α and ‐δ expressions in SAMP8 led to reduced binding between ACh and AChRs. As a compensatory mechanism, AChE activity was decreased to ensure sufficient ACh for binding with AChRs. However, increased ACh release would lead to the dispersion of AChRs cluster, which was also reported by other studies and this was related to a Cdk5‐dependent mechanism (Lin et al., 2005). All these alterations would contribute to NMJ functional degeneration at 8 months in SAMP8.

Mechanical vibrations applied to muscles could elicit a reflex muscle contraction named ‘tonic vibration reflex’. Thus, the excitatory inflow during vibration stimulation was mainly related to the reflex activation of NMJ (Cardinale & Bosco, 2003). LMHFV could reduce AChE activity at Months 2, 4, and 6 posttreatment. A previous study suggested that AChE inhibition may affect amyloid precursor protein processing and protect neurons against a variety of insults (Rees & Brimijoin, 2003). Blotnick et al. reported horizontal treadmill training could elevate AChE activity in fast muscles of adult rats (Blotnick & Anglister). In contrast, resistance exercise could reduce AChE activity in rats with Alzheimer's disease (Farzi et al., 2019). Hence, the effects of exercises on AChE activity were dependent on training types and physical conditions. At Month 2 posttreatment, reduced AChE activity and increased AChR α and δ subunits enhanced the binding between ACh and AChRs. This is because α and δ subunits constitute the binding site with ACh (Green & Wanamaker, 1998). Although LMHFV showed no significant effects on NMJ occupancy, fragmentation and discontinuity, NMJ function assessed by intra‐tetanic fatigue was still improved at Month 2 posttreatment. At Month 4 posttreatment, suppressed AChE activity and attenuated NMJ morphological degeneration enhanced the endplate excitability, which was accompanied by increased Type IIb MHC composition and muscle force, contributing to the elevated grip strength of SAMP8. Reduced AChR α and δ subunits expressions could not ensure enough binding between ACh and AChR, which may explain the not improved NMJ function assessed by neurotransmission failure and intra‐tetanic fatigue at Month 4 posttreatment. At Month 6 posttreatment, the positive effects of LMHFV on NMJ morphology and AChE activity suppression persisted and no differences of AChR α and δ subunits expressions were observed at this time point. So NMJ function improvement was persisted at Month 6 posttreatment. However, LMHFV showed no more elevated effects on MHC composition and muscle force, explaining the insignificant difference of grip strength at Month 6 posttreatment.

Rapsyn expressions decreased with aging, leading to morphological degeneration of postsynapse in SAMP8. On the other hand, Dok7 and Rapsyn were generally increased in SAMR1 with increasing age. Aare et al.'s (2016) study also reported that old rats presented higher expression of Rapsyn than young rats, while sarcopenic mice (neurotrypsin over‐expression) showed decreased level of Rapsyn compared with the wild type. Activated MuSK could recruit Dok7 and lead to the anchoring of AChRs by Rapsyn (Burden et al., 2018). Besides, MuSK was increased in myotubes with Dok7 knocked down (*p* < 0.01) (Figure S8A). So increased MuSK could be considered as a compensatory mechanism for decreased Dok7 and Rapsyn in SAMP8. Anagnostou et al. also reported that increased MuSK expression was a marker of denervation with aging (Anagnostou & Hepple, 2020). In contrast, during normal aging, increased Dok7 and Rapsyn was able to antagonize the loss of muscle mass and strength and as its upstream factor, MuSK expression was relatively suppressed in SAMR1.

LMHFV could significantly increase the protein expressions of Dok7 and Rapsyn at Month 4 posttreatment in SAMP8, which might lead to the improvements of AChRs cluster structure. Rapsyn was reported to be capable of inhibiting calpain activity, an enzyme involved in ACh‐mediated AChRs cluster dispersal (F. Chen et al., 2007). This could explain the attenuated dispersion of AChRs clustering at Month 4 post‐LMHFV treatment, although AChE activity was reduced. Hence, LMHFV could affect NMJ function in two distinct ways: reducing AChE activities and alleviating NMJ morphological degeneration by Dok7 and Rapsyn. Burden et al. (2013) reported Dok7, but not Rapsyn, was related to synapse‐specific gene expression. Besides, only Dok7 mRNA expression was increased at Month 4 posttreatment while in in vitro, knocking down Dok7 could reduce Rapsyn expression. So it was reasonable that LMHFV could increase the expression of Rapsyn by acting on Dok7.

LMHFV suppressed ERK1/2 phosphorylation at Month 4 posttreatment but presented no effects on the phosphorylation of p38. This was consistent with a previous study, revealing the effects of exercise on MAPKs depended on training types, in which low‐intensity or accustomed exercise collectively utilized ERK1/2 (Raney & Turcotte, 2006). Activated ERK1/2 was reported to inhibit myoblast differentiation (Knight & Kothary, 2011), explaining LMHFV could only suppress ERK1/2 phosphorylation and promote AChRs clustering when applied from D1 to D6 of myoblast differentiation. To further verify their relationship, blocking ERK1/2 phosphorylation was found to enhance AChRs clustering and increase Dok7 protein expression in vitro. In myotubes with ERK1/2 phosphorylation blocked, knocking down Dok7 could still reduce AChRs cluster formation, which could not be retarded by LMHFV. Taken altogether, LMHFV should increase the Dok7 expression through suppressing ERK1/2 phosphorylation.

Our study has some shortcomings. As human samples are precious and difficult to obtain, age or gender adjustment cannot be made due to limited sample size. The mice are quadrupedal, while humans are bipedal. As a result, they have different standing modes that may lead to different loading patterns on the vibration platform and different energy transference. This is also a challenge to expand on the different effects of LMHFV in in vivo and in vitro experiments when taking weight bearing into consideration. To maintain consistency, we applied the same vibration setting in both in vivo and in vitro experiments: 35 Hz, 0.3 g, 20 min. These settings are also consistent with our previous clinical trial (Leung et al., 2014), where we reported a significant positive effect on increasing muscle strength and reducing fall rate in community‐dwelling older adults. Additionally, further animal studies investigating Dok7 and ERK1/2 phosphorylation regulation were needed to solidify the conclusion that LMHFV could attenuate the progress of sarcopenia and NMJ degeneration by increasing Dok7 expression through suppressing ERK1/2 phosphorylation. Furthermore, our in vitro experiments did not include muscle–nerve coculture in in vitro experiment, we focused solely on myoblasts and AChRs. The immune system may be the potential mechanism; IL‐6 was reported to be implicated in the pathogenesis of sarcopenia and the binding of IL‐6 to IL‐6 receptor could initiate gp130‐dependent signaling pathway which would activate ERK1/2 (Fix et al., 2019; Nelke et al., 2019).

In conclusion, our observations revealed morphological differences in the NMJs of sarcopenic and non‐sarcopenic individuals. In the sarcopenic SAMP8 model, NMJ degeneration was found to be associated with the onset of sarcopenia. Our findings suggest that LMHFV could mitigate NMJ degeneration and sarcopenia progression by increasing Dok7 expression through suppressing ERK1/2 phosphorylation in skeletal muscle. Hence, mechanical stimulation targeting NMJ degeneration emerges as viable biophysical intervention to preserve neuromuscular health during aging.

## 4 MATERIALS AND METHODS

### 4.1 Patients

The research protocol for tissue sampling was approved by the Clinical Research Ethics Committee in the Chinese University of Hong Kong (Ref. 2021.008) and the Clinical Research Ethics Committee in Nanjing Drum Tower Hospital (Ref. AF/SC‐08/03.0). Patients with hip fracture receiving hip arthroplasty were recruited and grouped using AWGS 2019 definition and cutoffs (Chen et al., 2020). Inclusion criteria included (1) 60 years old or above; (2) both genders. Exclusion criteria included (1) chairbound or bedbound; (2) cognitive dysfunction with severe dementia, stroke with paralysis and infectious disease; (3) pathologic fracture. Grip strength and appendicular skeletal muscle mass index (ASMI) were measured following our previous protocols (Cheng et al., 2021). The patient information was seen in Table S1.

### 4.2 Experimental animals

Male C57BL/6 (3 and 24 months), SAMP8 (3, 6, 8, 10, and 12 months) and SAMR1 (3, 6, 8, 10, and 12 months) mice were obtained from the Laboratory Animal Service Center, the Chinese University of Hong Kong. The research protocol was approved by the Animal Experimentation Ethics Committee of the Chinese University of Hong Kong (Ref: 18/262/MIS). Measurements below were blinded with respect to treatment and type of mice.

### 4.3 LMHFV treatment

SAMP8 in vibration group (VIB) were applied with LMHFV treatment (20 min/day, 5 days/week) from 6 months old and continued until three specified time points (Months 2, 4, and 6 posttreatment). For example, Month 2 posttreatment means 6‐month‐old mice were applied with LMHFV treatment till 8‐month‐old (5 days/week for 2 months). SAMP8 in VIB group were housed in an individual and bottomless cage when receiving the vibration treatment (V‐Health Ltd, HKSAR, China). The LMHFV treatment protocol was the same as our previous studies (35 Hz, 0.3 *g* (*g* = gravitational acceleration), 0.1 mm vertical displacement) (Guo et al., 2016; Wang et al., 2020). The mice in the control group (CTL) were also put on vibration platform with power off to receive sham treatment.

For in vitro experiments, the culture plate was placed directly on the vibration platform. The vibration settings were the same as in vivo study (35 Hz, 0.3 *g*).

### 4.4 Grip strength measurement

Handgrip strength of patients was measured by a dynamometer (5030JI, JAMAR, Bolingbrook, IL, USA) on the dominant hand. Grip strength of mice was measured with a force gauge (Mark‐10 Corporation, USA) (Takeshita et al., 2017). The maximum strength was taken from three attempts.

### 4.5 Skeletal muscle mass measurement in patients

Total appendicular skeletal muscle mass (ASM) by dual energy x‐ray absorptiometry (DXA, Horizon, Hologic, Marlborough, MA, USA) was evaluated by segmented measurement of muscle mass at four limbs (Cheng et al., 2021). The ASM was then adjusted to the square of height to calculate the ASMI (kg/m^2^).

### 4.6 Ex vivo skeletal muscle and NMJ functional test

Details seen in Figures S1 and S2.

### 4.7 Histological and immunofluorescence analysis

Details seen in Figure S3.

### 4.8 Gene expression analysis by Western blot analysis and real‐time quantitative RT‐PCR

Proteins of skeletal muscles and cells were harvested and digested for western blot analysis according to our previous protocol (Wang et al., 2020). Primary and secondary antibodies used seen in Supplementary materials.

Total RNA of skeletal muscles and cells was harvested for real‐time quantitative RT‐PCR following our previous protocol (Wang et al., 2020). Gene‐specific primers were included in Table S2.

### 4.9 Acetylcholinesterase (AChE) activity assay

AChE activity was assessed with soluble proteins extracted from muscles following manufacturer's instruction (ab138871, Abcam, UK).

### 4.10 Isolation of primary myoblasts

Details seen in Figure S4.

### 4.11 Knocking down Dok7 and blocking ERK1/2 phosphorylation in vitro

The shRNA sequence against mouse Dok7 and scrambled negative control sequence were the same as previous studies (Eguchi et al., 2016). Plasmids were transfected into myoblasts by Lipofectamine 3000 Reagent (Thermo Scientific, Waltham, USA). PD0325901 (1.5 μg/mL) (Sigma‐Aldrich, USA) was used to block ERK1/2 phosphorylation in vitro.

### 4.12 Myoblast viability assay

Details seen in Figure S7.

### 4.13 Statistics

SPSS (IBM Corp, NY, USA) and GraphPad Prism (Graphpad Software, CA, USA) were used for statistical analyses. All quantitative data were expressed as mean ± standard deviation. Statistical comparisons were performed using two‐way repeated measures ANOVA, one‐way ANOVA followed by post hoc Bonferroni, LSD or Tamhane's T2 tests, Student's *t*‐test, as indicated in results and figure legends. Result summary of two‐way repeated measures ANOVA analyses were presented in Table S3. Significance was set at *p* < 0.05.

## AUTHOR CONTRIBUTIONS

ZB, SK‐HC, LQ, and W‐HC conceived and designed the project. W‐HC, QJ, LQ, and SK‐HC obtained the funding. ZB and CC performed all experiments. CL and SC helped to evaluate sarcopenia status of recruited patients. YL helped to perform western blot. SK‐HC, CR, RMYW, LQ, and W‐HC gave input to data analyses. RMYW and ZX collected clinical muscle biopsies. ZB, SK‐HC, and W‐HC wrote the manuscript. W‐HC, SK‐HC, CR, BHKY, QJ, and LQ revised the manuscript. CR is an author of several issued and pending patents related to the use of low‐intensity vibration for the treatment of musculoskeletal injury and disease.

## FUNDING INFORMATION

This work was supported by Collaborative Research Fund (Ref: C4032‐21GF), General Research Grant (Ref: 14114822), Group Research Scheme (Ref: 3110146), Health Care and Promotion Scheme (Ref: 02180118), Area of Excellence (Ref: AoE/M‐402/20), National Basic Research Program of China (Ref: 2021YFA1201404), Science and Technology Cooperation Program (Ref: 202308002), and Major Project of NSFC (Ref: 81991514).

## CONFLICT OF INTEREST STATEMENT

The authors have declared that no conflict of interest exists.

## Supporting information


Appendix S1

## Data Availability

The data that support the findings of this study are available from the corresponding author upon reasonable request.
