# Supplementary material for: Prevention of age‐related neuromuscular junction degeneration in sarcopenia by low‐magnitude high‐frequency vibration
Source: Aging Cell. 2024 Mar 27;23(7):e14156. doi: 10.1111/acel.14156 (PMC11258441; doi:10.1111/acel.14156)
Supplement: Supplementary file 1 — Appendix S1 [file ACEL-23-e14156-s001.docx]

***Ex vivo* skeletal muscle and NMJ functional test**

The skeletal muscle and NMJ functional test were performed by the *ex vivo* muscle functional test system (800A, Aurora Scientific Inc, Newmarket, Canada) according to previous established methods (**Figure S1A**) ([Aldrich, Shander, Chaudhry, & Nagashima, 1986](#_ENREF_1); [Personius & Sawyer, 2006](#_ENREF_6); [Pratt et al., 2015](#_ENREF_8); [Rizzuto, Pisu, Musaro, & Del Prete, 2015](#_ENREF_9)). To preserve intact nerve terminals and NMJ structure well, triceps surae-sciatic nerve was used to test muscle and NMJ function. To reduce the excitatory phase of anesthesia, a brief anesthesia was induced with isoflurane, followed with a long-lasting anesthesia induced and maintained by intraperitoneal injections of Na+ pentobarbital (1.5 mg/mouse) ([Jensen et al., 2018](#_ENREF_3)).

To increase the chance of detecting a deficit in NMJ function, optimal stimulation frequency and current were needed to maximally stress the neuromuscular system. SAMP8 at 6 months old were used to confirm the optimum parameter values. Three different frequencies were tried to trigger triceps surae contraction. 50Hz was found optimal to induce the maximal contraction by stimulating muscle and sciatic nerve. At 80Hz of nerve stimulation, there was an obvious slope at the platform of the oscillograph, indicating this frequency was too high in nerve tetanic stimulation. As a result, 50Hz was determined as the optimum frequency of stimulation (**Figure S1B**). To confirm the optimal stimulation current value, 100mA, 300mA and 1000mA were used to induce triceps surae contraction by directly stimulating the muscle and 300mA was found appropriate to trigger the maximal contraction. Therefore, 300mA was determined as the optimum current to stimulate the muscle directly (**Figure S1C**). 1mA, 5mA, 10mA and 100mA were applied to induce triceps surae contraction by stimulating the sciatic nerve and 5mA was able to trigger the maximum muscle contraction. Hence, 5mA was determined as the optimum current to stimulate the sciatic nerve (**Figure S1D**). To confirm the optimal stimulation pulse width, 0.2ms, 0.5ms, 0.8 ms and 1.0ms were used to induce triceps surae twitch contraction by directly stimulating the muscle or the sciatic nerve. To better protect NMJ function during muscle stimulations, we chose 0.2ms as the optimum pulse width for muscle. Furthermore, to fully activate NMJ and observe NMJ functional differences more easily, we chose 0.8ms as the optimum pulse width for nerve (**Figure S1E**).


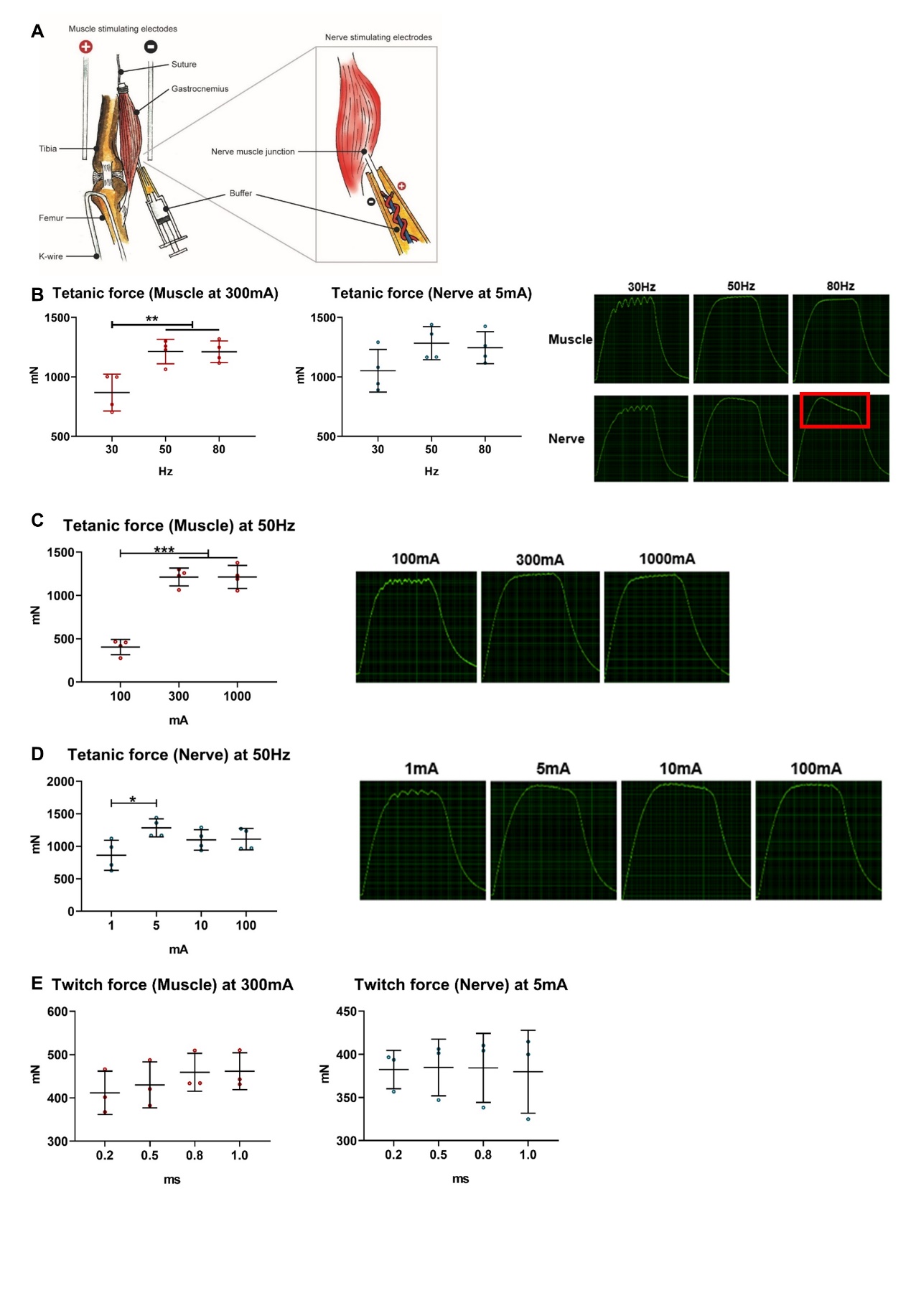


**Figure S1** Validating the optimal stimulation frequency and current intensity in *ex vivo* NMJ and muscle function test.

**(A)** Apparatus for assessing *ex vivo* NMJ and muscle function test. Two parallel electrodes can stimulate muscle contraction directly. The suction electrode can induce muscle contraction indirectly by stimulating the sciatic nerve. **(B)** n=4. 50Hz was optimal to induce the maximal contraction by stimulating muscle and sciatic nerve and a significant slope at the platform was observed in the oscillograph of nerve stimulation at 80Hz. **(C)** n=4. 300mA was sufficient to trigger the maximal contraction by directly stimulating the muscle and no obvious differences of the platform in the oscillograph were observed between 300mA and 1000mA of muscle stimulations. **(D)** n=4. 5mA was sufficient to trigger the maximum muscle contraction by stimulating the sciatic nerve and no obvious differences of the platform in the oscillograph were observed among 5mA, 10mA and 1000mA of nerve stimulations. **(E)** n=3. In twitch stimulation, no significant difference of twitch force were observed in 0.2ms, 0.5ms, 0.8 ms and 1.0ms of pulse width by stimulating muscle or sciatic nerve. **^*^**p<0.05, ^**^p < 0.01, ^***^p < 0.001 in Bonferroni post-hoc test following one-way ANOVA.

Under the optimal length (L0), the muscle and nerve were stimulated separately by a single stimulus with 1 min interval to assess the twitch characteristic (muscle stimulus: 300mA, 0.2ms pulse width; nerve stimulus: 5mA, 0.8ms pulse width). After 1 min, the muscle and nerve were electronically stimulated separately by a tetanic stimulus with 2 min interval to evaluate the tetanic characteristic (muscle stimulus: 300mA, 300ms duration, 0.2ms pulse width, 50Hz stimulation frequency; nerve stimulus: 5mA, 300ms duration, 0.8ms pulse width, 50Hz stimulation frequency). Two separate consecutive 100 cycles with a rest time of 0.7s each of tetanic stimulus of only direct muscle stimulation or sciatic nerve stimulation at 50Hz were performed to investigate fatigue characteristics. The order of stimulation was randomized (muscle or sciatic nerve) for each sample and a 15 min rest was used between trials (**Figure S2**). Neurotransmission failure and intra-tetanic fatigue of muscle and NMJ could be generated from the two separate consecutive tetanic pulse trainings ([Personius & Sawyer, 2006](#_ENREF_6)).

After completing the *ex vivo* functional test, CSA was calculated by dividing the wet weight of triceps surae muscle by L0 and the density of mammalian skeletal muscle (1.06mg/mm^3^).


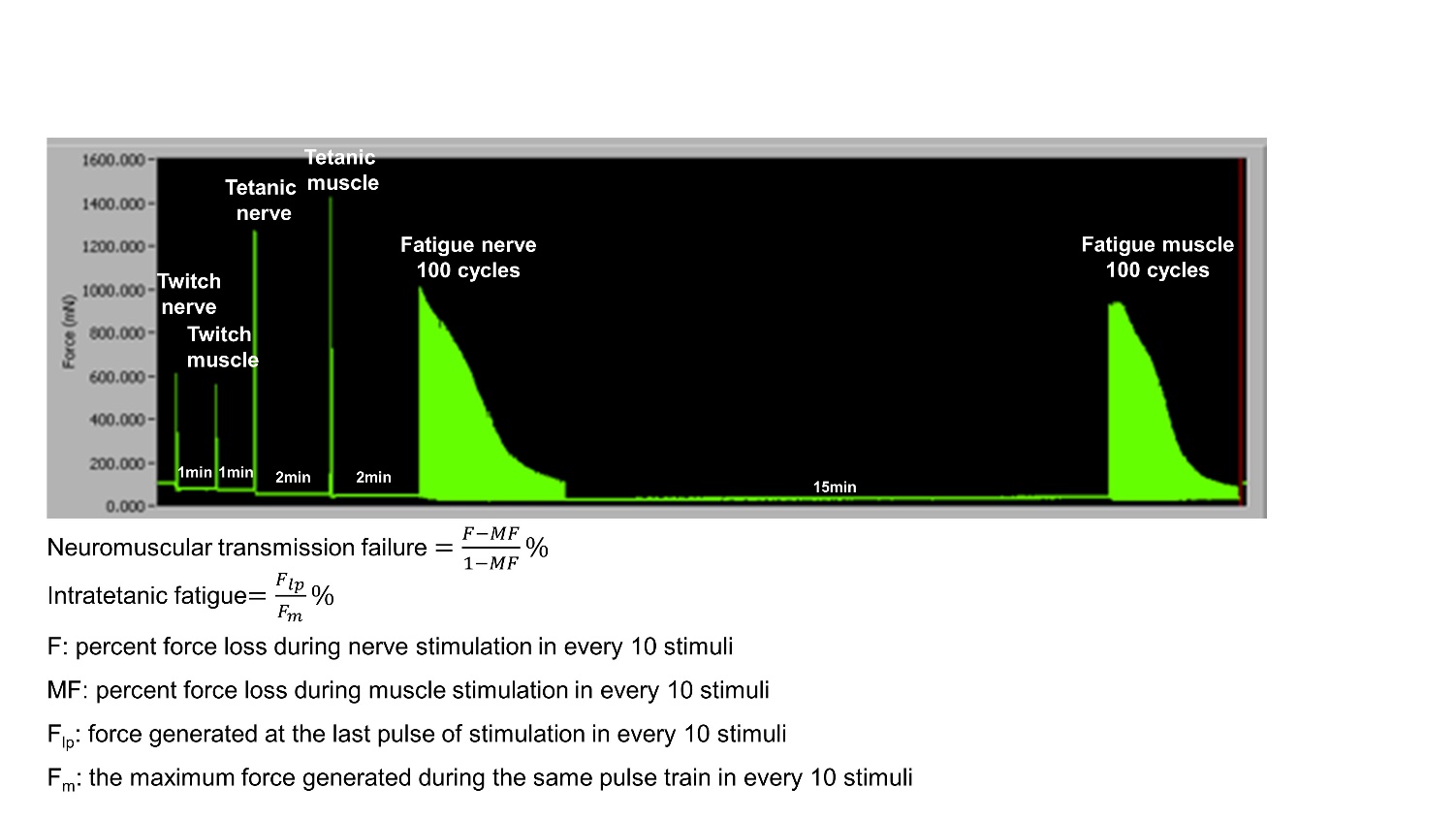


**Figure S2** *Ex vivo* skeletal muscle and NMJ functional test diagram.

**Histological and immunofluorescence analysis**

Gastrocnemius of mice were cryo-sectioned for histological examination. Muscle samples sectioned at 10μm thick were subjected to Dok7 staining for evaluation of Dok7 distribution along with AChRs and MHC staining for muscle fiber typing based on our previous protocol ([Wang et al., 2020](#_ENREF_13)). Primary antibodies against AChRs (α-bungarotoxin (α-BTX), Alexa Fluro 555 conjugate (B35451 at 1:200, Thermo scientific, USA) and Dok7 (PA5-95620 at 1:100, Thermo scientific, USA) were mixed to form the primary antibody cocktail. Secondary antibody Alexa Fluor 488 (A27034, Thermo scientific, USA) was diluted at 1:1000. Digital images of Dok7 along with AChRs were taken with a confocal laser-scanning microscope (Carl Zeiss LSM 880) at 63x magnification. Primary antibodies against MHC I (BA-F8, Developmental Studies Hybridoma Bank, USA), MHC IIa (SC-71, Developmental Studies Hybridoma Bank, USA) and MHC IIb (BF-F3, Developmental Studies Hybridoma Bank, USA) were mixed to form the primary antibody cocktail (4μg/ml). Secondary antibodies including Alexa Fluor 350 IgG2b, Alexa Fluor 488 IgG1 and Alexa Fluor 555 IgM (4μg/ml, Thermo scientific, USA) were diluted and mixed to form the secondary antibody cocktail. Slides were visualized with a fluorescence microscope (Leica, DM 6000B upright microscope, Leica-microsystem, GmbH, Werzlar, Germany) at 20x magnifications.

Whole mount extensor digitorum longus (EDL) of mice was stored in 4% PFA at 4℃ for a minimum of 24h prior to NMJ staining according to previous protocols ([Jones et al., 2016a](#_ENREF_4); [Pratt, Iyer, Shah, & Lovering, 2018](#_ENREF_7)). Primary antibodies against AChRs (B35451 at 1:200, Thermo scientific, USA), synaptophysin (MA1-213 at 1:200, Thermo scientific, USA) and neurofilament (837904 at 1:100, Biolegend, USA) were mixed to form the primary antibody cocktail. Secondary antibody Alexa Fluor 488 (A21121, Thermo scientific, USA) was diluted at 1:500. Digital images of NMJ from whole EDL were taken with a confocal laser-scanning microscope (Carl Zeiss LSM 880) at 63x magnification. Only *en face* NMJ images were used for further analysis with Image J software (NIH, Bethesda, USA).

NMJ morphology was analysed based on previous studies ([Jones et al., 2016b](#_ENREF_5)). In brief, using Image J software, binary images, endplate images and skeleton images were generated from original images (**Figure S3A**). From binary images, “Fragmentation”, “AChR cluster area” and “Occupancy” were quantified. “Fragmentation” was calculated as *1-(1/number of AChR islands)*. “Occupancy” was calculated as *(Nerve terminal area/AChR cluster area) in %*. From endplate images, “Endplate area” was quantified. “Compactness” was calculated as *(AChR cluster area/Endplate area) in %*. From skeleton images, “Branching” was calculated as the number of pixels which with three neighbors. “Discontinuity” was calculated as *the number of pixels which are at the end of a line of pixels with one neighbor*.


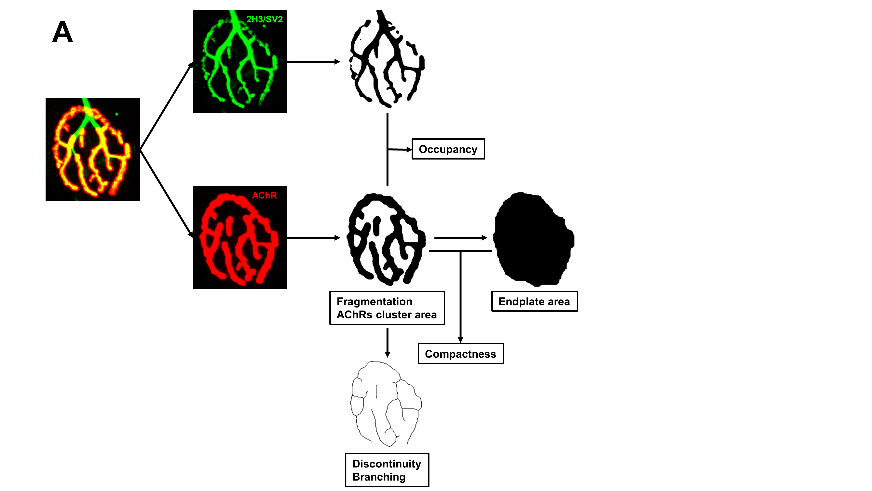


**Figure S3** Overview of the NMJ morphology analyses

**(A)** Flowchart demonstrating the sequence of morphological analyses for NMJ.

For the evaluation of AChRs cluster formation *in vitro*, fully differentiated myotubes were incubated with Alexa Fluro 555 conjugated α-BTX (B35451, 1:500 at differentiation medium, Thermo scientific, USA) for 1 h at 37℃ in dark. Cells were then fixed with 4% PFA and mounted with Prolong Gold antifade reagent with DAPI (Invitrogen, CA, USA). The stained cells were analyzed with a confocal laser-scanning microscope (Carl Zeiss LSM 880) at 40x magnification. AChR clusters were quantified using Image J software and only AChR clusters with area >10 μm^2^ were counted.

Human vastus lateralis muscle biopsies were cryo-sectioned for immunofluorescence analysis. Muscle samples were sectioned at 10μm thick. Primary antibodies against AChRs (B35451 at 1:200, Thermo scientific, USA), SV2 (Synaptic vesicle glycoprotein 2A at 1:200, DSHB, USA) and 2H3 (neurofilament (NF-M) at 1:100, DSHB, USA) were mixed to form the primary antibody cocktail. Secondary antibody Alexa Fluor 488 (A21121, Thermo scientific, USA) was diluted at 1:500. Digital images of NMJ from muscle section were taken with a confocal laser-scanning microscope (Carl Zeiss LSM 880) at 100x magnification. Only *en face* NMJ images were used for further analysis with Image J software.

**Primary and secondary antibodies used in Western blot analysis**

GAPDH (1:2000, MA5-15738, Invitrogen, USA)

MuSK (1:2000, ab92950, Abcam, UK)

Dok7 (1:2000, A9537, ABclonal, USA)

Rapsyn (1:2000, A6716, ABclonal, USA)

ERK1/2 (1:2000, 4695s, Cell Signaling Technology, USA)

p-ERK1/2 (1:2000, 8544s, Cell Signaling Technology, USA)

p38 (1:2000, 9212, Cell Signaling Technology, USA)

p-p38 (1:2000, 9216s, Cell Signaling Technology, USA)

Anti-rabbit IgG, HRP-linked antibody (1:5000, Cell Signaling Technology, USA)

Goat anti-mouse IgG (H+L) antibody (1:5000, Invitrogen, USA).

**Isolation of primary myoblasts**

To investigate the relationship of LMHFV, NMJ degeneration, sarcopenia, Dok7 and ERK1/2 *in vitro*, primary myoblasts were isolated from skeletal muscle tissues of SAMP8 according to previous protocols ([Hindi, McMillan, Afroze, Hindi, & Kumar, 2017](#_ENREF_2); [Shahini et al., 2018](#_ENREF_11); [Soriano-Arroquia, Clegg, Molloy, & Goljanek-Whysall, 2017](#_ENREF_12)).

Tibial anterior muscle (TA), EDL and gastrocnemius from both hind limbs were dissected and minced in PBS. After removing the supernatant, muscle tissues were incubated in collagenase II-CaCl2 solution (400U/mL of collagenase II and 2.5mM CaCl2 in ultra-pure water) at 37°C for 60 min. Digested tissues were centrifuged at 443×g for 3 min at room temperature and the supernatant was removed. 0.25% trypsin was added and incubated with tissues at 37°C for 30 min. Muscle solution was filtered through a 70μm strainer and centrifuged at 443×g for 5 min to pellet the cells which were dissolved into growth medium (20% FBS, 10% non-heat inactivated horse serum (nh-HS), 1% penicillin-streptomycin-neomycin (PSN), 1% α-glutamine and 2.5ng/mL of recombinant human basic fibroblast growth factor (bFGF) in F12 medium). Cellular mixtures were seeded onto the Matrigel pre-coated T25 flask and kept in the incubator at 37°C for 72 h. After 72 h, growth medium was changed every two days until 70% confluence. Adherent cells were detached by 0.05% trypsin and collected with 5ml growth medium. Mixed cells were transferred onto non-coated T25 flask and placed at 37°C for 1 h. Supernatant was transferred to a new Matrigel pre-coated T25 flask and marked as P1. Pax7 and MyoD are highly expressed in myoblasts ([Rudnicki, Le Grand, McKinnell, & Kuang, 2008](#_ENREF_10)). With the co-staining of nucleus (DAPI, Invitrogen, CA, USA), Pax7 (ab199010 at 1:200, Abcam, UK, with Alexa Fluor 488 (A21121, Thermo scientific, USA) at 1:500) and MyoD (ab203383 at 1:100, Abcam, UK, with Alexa Fluor 555 (A21428, Thermo scientific, USA) at 1:500), the purity of myoblasts could be up to 98±2% (**Figure S4A**). To obtain good quality of myotubes, differentiation medium (2% nh-HS in low-glucose DMEM medium) was added when myoblasts reached 85–95% confluence.


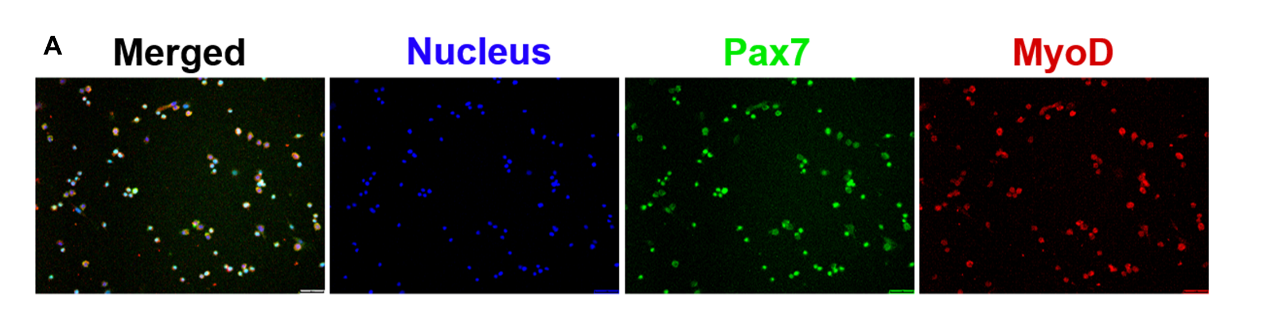


**Figure S4** Myoblast purity assessment

**(A)** n=10 fields. With the co-staining of nucleus, Pax7 and MyoD, the purity of myoblasts was up to 98±2%.


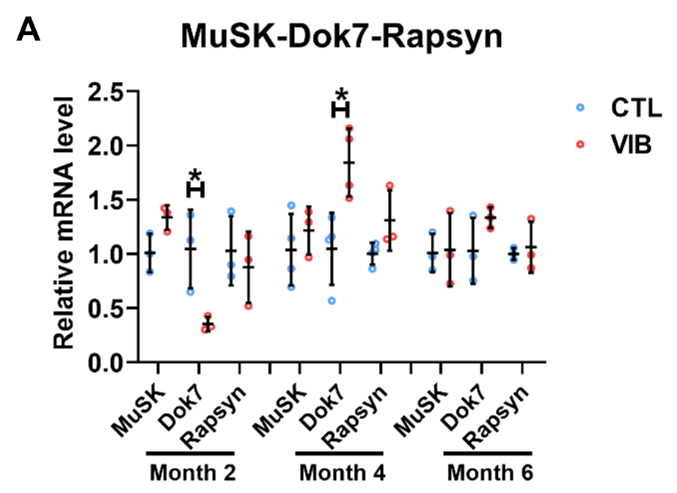


**Figure S5** mRNA expressions of MuSK-Dok7-Ranpsyn in CTL and VIB groups at month 2, 4 and 6 post-treatment.

**(A)** mRNA expressions of MuSK, Dok7 and Rapsyn at month 2, 4 and 6 post-treatment (8, 10 and 12 months old, n=3-4). **^*^**p<0.05 (CTL vs VIB at each timepoint) in Student’s t-test.


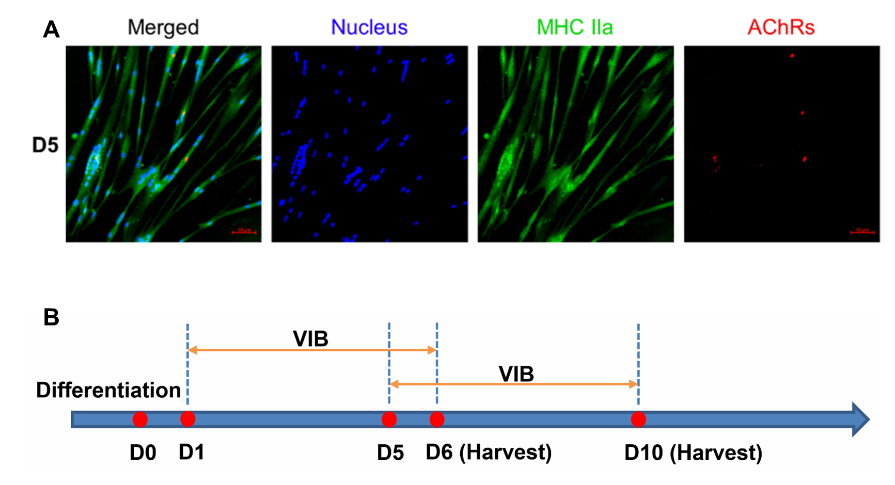


**Figure S6** Myotube formation assessment.

**(A)** Mature myotubes were observed to form at D5 of differentiation with co-staining of nucleus, MHC IIa and AChRs. **(B)** LMHFV was applied in two different schemes: D1 to D6 or D5 to D10 of myoblast differentiation.

**Myoblast viability assay**

The viability of myoblasts was measured using the Cell Counting Kit-8 (CCK-8) assay. Normal and Dok7 knocked-down myoblasts were seeded in 96-well plates at 2,000 cells in 100μl per well. After 48 hours, 10μl CCK-8 solution was added to each well and incubated for 4 hours. The absorbance at 450nm was read using a microplate reader (MCE, USA). Dok7 knock-down presented no significant effects on absorbance at 450nm (**Figure S7**).


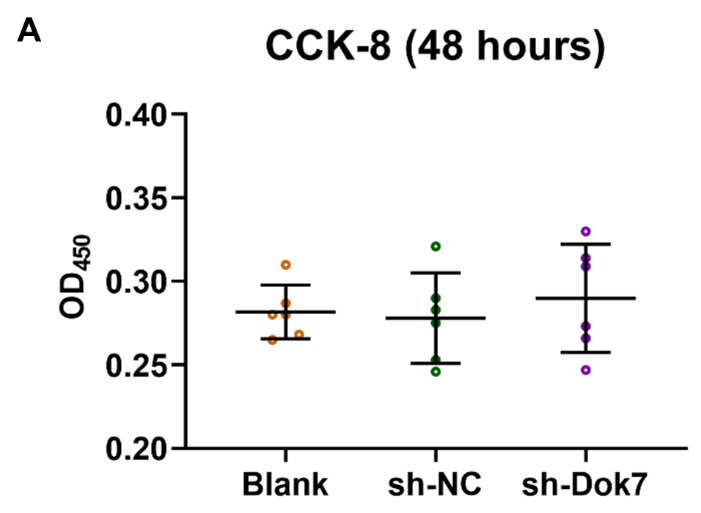


**Figure S7** Effects of Dok7 knock-down on the viability of myoblasts.

**(A)** Cytotoxic effects of Dok7 knock-down on myoblasts after 48 hours in the CCK-8 assay (n=6).


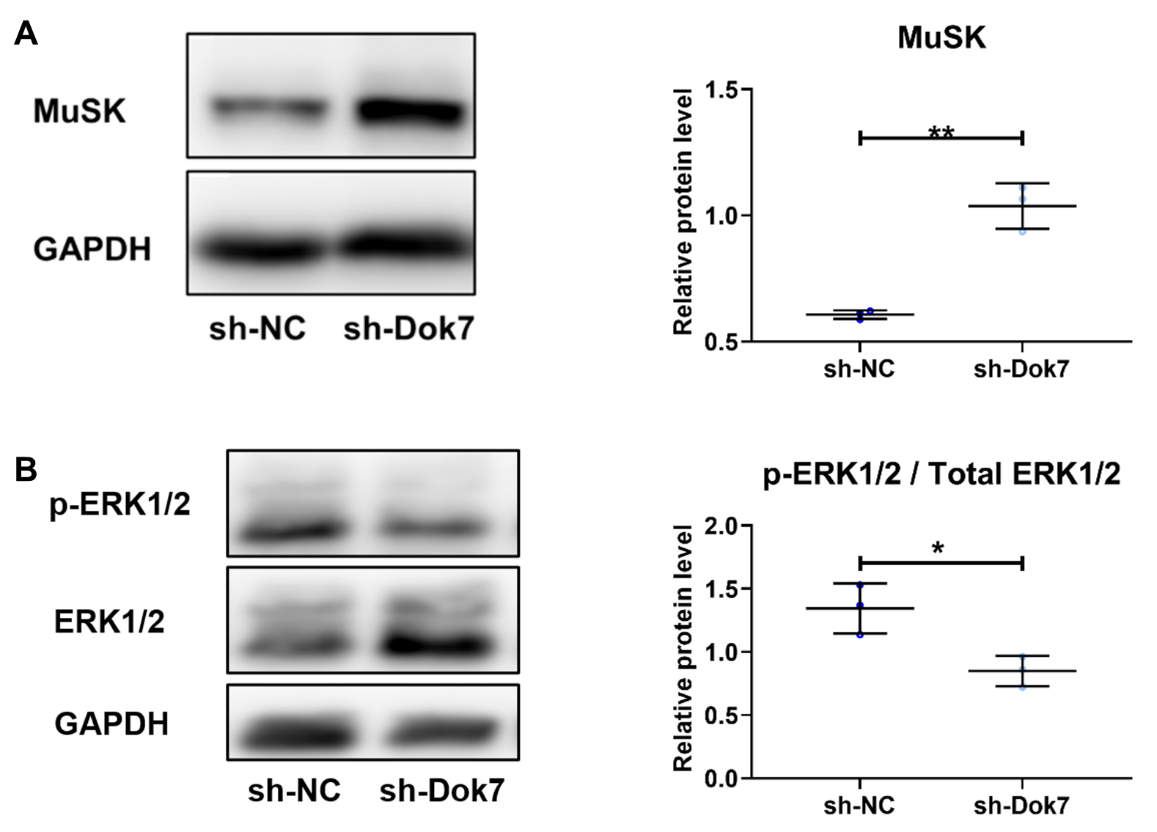


**Figure S8** MuSK and ERK1/2 phosphorylation expressions in myotubes with Dok7 knocked down.

**(A)** Western blot results of MuSK and GAPDH at D6 of myoblast differentiation in sh-NC and sh-Dok7 groups (n=3). **(B)** Western blot results of p-ERK1/2, ERK1/2 and GAPDH at D6 of myoblast differentiation in sh-NC and sh-Dok7 groups (n=3). **^*^**p<0.05, **^**^**p<0.01 in Student’s t-test.

**Table S1** Patient information for the recruited muscle biopsy.

| **Patient ID** | **Sarcopenia status** | **Age (yr)** | **Gender** | **Height (cm)** | **Weight (Kg)** | **BMI** | **Comorbidities** |
| --- | --- | --- | --- | --- | --- | --- | --- |
| 1 | Non-sarco | 69 | Female | 162 | 65 | 24.77 | HT, FL |
| 2 | Non-sarco | 83 | Female | 148 | 47 | 21.46 | Hypothyroidism |
| 3 | Non-sarco | 65 | Female | 159 | 62 | 24.52 | HT |
| 4 | Non-sarco | 79 | Female | 160 | 56 | 21.88 | / |
| 5 | Sarco | 90 | Female | 155 | 37 | 15.40 | DM |
| 6 | Sarco | 87 | Male | 158 | 54 | 21.63 | HT, DM |
| 7 | Sarco | 74 | Female | 154 | 50 | 21.08 | HT |
| 8 | Sarco | 83 | Female | 139 | 25 | 12.94 | / |
| 9 | Sarco | 71 | Female | 155 | 50 | 20.81 | DM |

BMI: Body mass index; HT: Hypertension; FL: Fatty liver; DM: Diabetes mellitus.

**Table S2** Primer sequences for RT-PCR.

| **Gene** | **Sequence(5'to3')** |
| --- | --- |
| GAPDH | F: 5’-TGGCCTTCCGTGTTCCTAC-3’  R: 5’-GAGTTGCTGTTGAAGTCGCA-3’ |
| AChR-α | F: 5’-CTCTCGACTGTTCTCCTGCTG-3’  R: 5’-GTAGACCCACGGTGACTTGTA-3’ |
| AChR-δ | F: 5’-GAATGAGGAACAAAGGCTGATCC-3’  R: 5’-GGTGAGACTTAGGGCGACAT-3’ |
| MuSK | F: 5’-TACAGAGGGGAGGTGTGTGAT-3’  R: 5’-TCCCGGTAGGAGGTGTTGAA-3’ |
| Rapsyn | F: 5’-GGCAGGACCAGACAAAGCAA-3’  R: 5’-CGAGTGAGCTGTTACCAAGCA-3’ |
| Dok7 | F: 5’-ATGCTGGTCTACAAGGACAAATG-3’  R: 5’-AGCTACTGTCACATGGAACCT-3’ |
| Atrogin-1 | F: 5’-CAGCTTCGTGAGCGACCTC-3’  R: 5’-GGCAGTCGAGAAGTCCAGTC-3’ |
| MuRF-1 | F: 5’-CCAGGCTGCGAATCCCTAC-3’  R: 5’- ATTTTCTCGTCTTCGTGTTCCTT-3’ |

**Table S3** Result summary of two-way repeated measures ANOVA analyses

| **Summary** | **Source** | **Intergroup (F, *p*)** | **Interaction (F, *p*)** | **Pairwise comparisons (Bonferroni adjustment)** |
| --- | --- | --- | --- | --- |
| NF at 10-months in SAMP8 and SAMR1 | Mouse strain * stimulus times | 5.317, 0.047 | 1.292, 0.299 | 0.047 |
| NF of SAMP8 at 3,6, 8, 10 and 12 months | Mouse age * stimulus times | 5.416, 0.003 | 2.281, 0.000 | Between 6 and 8 months: p<0.05 at 70^th^, 80^th^ and 90^th^ stimulus and p<0.01 at 50^th^, 60^th^ and 100^th^ stimulus; Between 6 and 12 months: p<0.05 at 60^th^ and 70^th^ stimulus, p<0.01 at 80^th^, 90^th^ and p<0.001 at 100^th^ stimulus |
| IF (muscle) of SAMP8 at 3,6, 8, 10 and 12 months | Mouse age * stimulus times | 1.134, 0.366 | 2.561, 0.001 | p<0.05 at 10^th^ stimulus between 3 and 6 months; p<0.01 at 10^th^ stimulus between 3 and 8 months and p<0.001 at 10^th^ stimulus between 3 and 10 months |
| IF (nerve) of SAMP8 at 3,6, 8, 10 and 12 months | Mouse age * stimulus times | 3.837, 0.016 | 0.503, 0.961 | p<0.05 between 6 and 8 months and p<0.01 between 6 and 12 months |
| NF at month 2 | LMHFV * stimulus times | 2.95, 0.124 | 1.102, 0.345 | NS |
| NF at month 4 | LMHFV * stimulus times | 0.466, 0.51 | 1.582, 0.236 | NS |
| NF at month 6 | LMHFV * stimulus times | 5.223, 0.048 | 0.978, 0.368 | 0.048 |
| IF (muscle) at month 2 | LMHFV * stimulus times | 0.004, 0.951 | 0.562, 0.566 | NS |
| IF (nerve) at month 2 | LMHFV * stimulus times | 7.428, 0.026 | 0.72, 0.452 | 0.026 |
| IF (muscle) at month 4 | LMHFV * stimulus times | 0.234, 0.639 | 1.009, 0.374 | NS |
| IF (nerve) at month 4 | LMHFV * stimulus times | 0.289, 0.603 | 0.841, 0.458 | NS |
| IF (muscle) at month 6 | LMHFV * stimulus times | 0.001, 0.979 | 1.065, 0.364 | NS |
| IF (nerve) at month 6 | LMHFV * stimulus times | 7.756, 0.021 | 1.905, 0.112 | 0.021 |

NF: Neurotransmission failure; IF: Intra-tetanic fatigue; LMHFV: Low-magnitude high-frequency vibration; NS: No sifnificant difference

**References**

Aldrich, T. K., Shander, A., Chaudhry, I., & Nagashima, H. (1986). Fatigue of isolated rat diaphragm: role of impaired neuromuscular transmission. *J Appl Physiol (1985), 61*(3), 1077-1083. doi:10.1152/jappl.1986.61.3.1077

Hindi, L., McMillan, J. D., Afroze, D., Hindi, S. M., & Kumar, A. (2017). Isolation, Culturing, and Differentiation of Primary Myoblasts from Skeletal Muscle of Adult Mice. *Bio Protoc, 7*(9). doi:10.21769/BioProtoc.2248

Jensen, D. B., Stecina, K., Wienecke, J., Hedegaard, A., Sukiasyan, N., Hultborn, H. R., & Meehan, C. F. (2018). The Subprimary Range of Firing Is Present in Both Cat and Mouse Spinal Motoneurons and Its Relationship to Force Development Is Similar for the Two Species. *J Neurosci, 38*(45), 9741-9753. doi:10.1523/JNEUROSCI.2898-17.2018

Jones, R. A., Reich, C. D., Dissanayake, K. N., Kristmundsdottir, F., Findlater, G. S., Ribchester, R. R., . . . Gillingwater, T. H. (2016a). NMJ-morph reveals principal components of synaptic morphology influencing structure-function relationships at the neuromuscular junction. *6*(12).

Jones, R. A., Reich, C. D., Dissanayake, K. N., Kristmundsdottir, F., Findlater, G. S., Ribchester, R. R., . . . Gillingwater, T. H. (2016b). NMJ-morph reveals principal components of synaptic morphology influencing structure-function relationships at the neuromuscular junction. *Open Biol, 6*(12). doi:10.1098/rsob.160240

Personius, K. E., & Sawyer, R. P. (2006). Variability and failure of neurotransmission in the diaphragm of mdx mice. *Neuromuscul Disord, 16*(3), 168-177. doi:10.1016/j.nmd.2006.01.002

Pratt, S. J. P., Iyer, S. R., Shah, S. B., & Lovering, R. M. (2018). Imaging Analysis of the Neuromuscular Junction in Dystrophic Muscle. *Methods Mol Biol, 1687*, 57-72. doi:10.1007/978-1-4939-7374-3_5

Pratt, S. J. P., Shah, S. B., Ward, C. W., Kerr, J. P., Stains, J. P., & Lovering, R. M. (2015). Recovery of altered neuromuscular junction morphology and muscle function in mdx mice after injury. *Cell Mol Life Sci, 72*(1), 153-164. doi:10.1007/s00018-014-1663-7

Rizzuto, E., Pisu, S., Musaro, A., & Del Prete, Z. (2015). Measuring Neuromuscular Junction Functionality in the SOD1(G93A) Animal Model of Amyotrophic Lateral Sclerosis. *Ann Biomed Eng, 43*(9), 2196-2206. doi:10.1007/s10439-015-1259-x

Rudnicki, M. A., Le Grand, F., McKinnell, I., & Kuang, S. (2008). The molecular regulation of muscle stem cell function. *Cold Spring Harb Symp Quant Biol, 73*, 323-331. doi:10.1101/sqb.2008.73.064

Shahini, A., Vydiam, K., Choudhury, D., Rajabian, N., Nguyen, T., Lei, P., & Andreadis, S. T. (2018). Efficient and high yield isolation of myoblasts from skeletal muscle. *Stem Cell Res, 30*, 122-129. doi:10.1016/j.scr.2018.05.017

Soriano-Arroquia, A., Clegg, P. D., Molloy, A. P., & Goljanek-Whysall, K. (2017). Preparation and Culture of Myogenic Precursor Cells/Primary Myoblasts from Skeletal Muscle of Adult and Aged Humans. *J Vis Exp*(120). doi:10.3791/55047

Wang, J., Cui, C., Chim, Y. N., Yao, H., Shi, L., Xu, J., . . . Cheung, W. H. (2020). Vibration and β-hydroxy-β-methylbutyrate treatment suppresses intramuscular fat infiltration and adipogenic differentiation in sarcopenic mice. *J Cachexia Sarcopenia Muscle, 11*(2), 564-577. doi:10.1002/jcsm.12535
